# The Iceman’s lithic toolkit: Raw material, technology, typology and use

**DOI:** 10.1371/journal.pone.0198292

**Published:** 2018-06-20

**Authors:** Ursula Wierer, Simona Arrighi, Stefano Bertola, Günther Kaufmann, Benno Baumgarten, Annaluisa Pedrotti, Patrizia Pernter, Jacques Pelegrin

**Affiliations:** 1 Soprintendenza Archeologia, Belle Arti e Paesaggio per la città metropolitana di Firenze e le province di Pistoia e Prato, Firenze, Italy; 2 Dipartimento di Scienze Fisiche, della Terra e dell’Ambiente, UR Preistoria e Antropologia, Università degli Studi di Siena, Siena, Italy; 3 Dipartimento di Studi Umanistici, Università degli Studi di Ferrara, Ferrara, Italy; 4 Institut für Geologie und Paläontologie, AG Hochgebirgsarchäologie und Quartärökologie, Universität Innsbruck, Innsbruck, Austria; 5 Südtiroler Archäologiemuseum/Museo Archeologico dell’Alto Adige, Bozen/Bolzano, Italy; 6 Naturmuseum Südtirol/Museo di Scienze Naturali dell’Alto Adige, Bozen/Bolzano, Italy; 7 Dipartimento di Lettere e Filosofia, Università degli Studi di Trento, Trento, Italy; 8 Department of Radiodiagnostics, Central Hospital Bolzano, Bozen/Bolzano, Italy; 9 CNRS—UMR 7055 Préhistoire et Technologie, MAE, Université Paris Nanterre, Paris, France; Max Planck Institute for the Science of Human History, GERMANY

## Abstract

The Tyrolean Iceman, a 5,300-year-old glacier mummy recovered at the Tisenjoch (South Tyrol, Italy) together with his clothes and personal equipment, represents a unique opportunity for prehistoric research. The present work examines the Iceman’s tools which are made from chert or are related to chert working - dagger, two arrowheads, endscraper, borer, small flake and antler retoucher - and considers also the arrowhead still embedded in the shoulder of the mummy. The interdisciplinary results achieved by study of the lithic raw material, technology, use-wear analysis, CT analysis and typology all add new information to Ötzi‘s individual history and his last days, and allow insights into the way of life of Alpine Copper Age communities. The chert raw material of the small assemblage originates from at least three different areas of provenance in the Southalpine region. One, or possibly two, sources derive from outcrops in the Trentino, specifically the Non Valley. Such variability suggests an extensive provisioning network, not at all limited to the Lessini mountains, which was able to reach the local communities. The Iceman’s toolkit displays typological characteristics of the Northern Italian tradition, but also comprises features typical of the Swiss Horgen culture, which will come as no surprise in the toolkit of a man who lived in a territory where transalpine contacts would have been of great importance. Ötzi was not a flintknapper, but he was able to resharpen his tools with a medium to good level of skill. Wear traces reveal that he was a right-hander. Most instruments in the toolkit had reached their final stage of usability, displaying extensive usage, mostly from plant working, resharpenings and breaks. Evidently Ötzi had not had any access to chert for quite some time, which must have been problematic during his last hectic days, preventing him from repairing and integrating his weapons, in particular his arrows. Freshly modified blade tools without any wear suggest planned work which he never carried out, possibly prevented by the events which made him return to the mountains where he was killed by a Southern Alpine archer.

## 1. Introduction

It was on 19^th^ September 1991 when tourists crossing a glacier on the main Alpine ridge between the Schnalstal (Italy) and the Ötztal (Austria) made an exceptional discovery: on the Tisenjoch, at 3210 m a.s.l., the naturally mummified body of a fully equipped man emerged from the ice. This unique witness of the Copper Age, who died between 3370–3100 cal BC (4550±19 BP uncal,1σ) after having been shot by an arrow and was subsequently preserved in the ice, has opened new perspectives for prehistoric and anthropological research into the reconstruction of lifestyle and physical conditions of the Alpine inhabitants of the latter part of the 4^th^ millennium BC [1–10]. For archaeological research the discovery and the context of this 45 years old man, along with his clothing and personal equipment, mostly made from exceptionally well-preserved organic material, represent a unique case study.

The present research deals with the Iceman’s tools which are made from chert or are related to chert working: the dagger blade, the two arrowheads, the three unhafted chert tools and the retoucher. The study also considers the arrowhead still embedded in the Iceman’s left shoulder which most probably caused his decease [11]. The aim of the study is to investigate the story of the owner, Ötzi, in order to gain insights into his individual history, last days and cultural and social background. All artefacts were found in the glacier gully near the Iceman. The belt pouch containing endscraper, borer and small flake was detected during the recovery of the corpse from the glacier on 23^rd^ September 1991. Its precise position is unknown but, judging from its good preservation, it very probably lay under the mummy [9, 12]. The belt poach contained also a bone awl and several pieces of *fomes fomentarius* fungus with traces of pyrite [9, 13, 14]. After the removal of the body, the retoucher and dagger came to light in the melt water. They lay in the immediate surroundings of the body [12]. However, the details as to where and how the bast scabbard recovered on the same day, was found are obscure. A few days after the official rescue a leather quiver was detected about 5 m north-east from the “mummy stone”, but several elements indicate that this was not its original position [12, 15]. The quiver contained two arrowheads separate from their broken shafts, 12 unfinished arrow shafts, a bundle of four antler points wrapped with bast, a single antler point, a bast cord and a bundle of sinews [9]. A shaft fragment, broken into three pieces, was found further away, near the copper axe, the back bag and the bow. It has been identified as the proximal fragment of the unfinished shaft 13 [15–16].

Thanks to the similarity of the copper axe, the dagger and the arrowheads with the grave goods of the Northern Italian Remedello Culture, the southern Alpine origin of the Iceman was hypothesized soon after the discovery [17–18]. His provenance from the Lower Vinschgau, the main valley at 1–2 days’ walk south from the discovery site, was then also indicated by the plant species of his equipment [16], and later confirmed by isotope analyses made on the mummy [19]. The Copper Age population of the Vinschgau is best attested by the site of Latsch, contemporary with the Iceman, settled by an agro-pastoral community [20]. Besides an economy centered on the valley floor, an increased interest in the mountain range, perceptible over the entire region during the Late Neolithic and Copper Age [21], can also be seen in the Vinschgau, where recent findings on passes at about 3,000 m a.s.l. also prove that the Alpine Ridge had been crossed during the 4^th^ and 3^rd^ millennium BC [22–23]. Unlike with other Alpine territories though, where mountain frequentation could be due to mountain pastoralism [24–25], no evidence for such activity has been found in the Schnals Valley before the 2^nd^ millennium BC [26–28]. The burial sites of the communities living in the Vinschgau have not been found. According to Dal Ri & Tecchiati [29] though, ancient findings of isolated lithic daggers could derive from destroyed burial sites, being daggers typical of grave goods of the period. The regional death rituals are characterized both by burials in rock-shelters and small caves, in cultural continuity with the Western Alpine Civate Culture, and by the deposition of the sometimes cremated bodies in large stone structures [30–31]. Significant testimonies to the ideological and religious world are the anthropomorphic stele [32–33], verified locally at Algund, Latsch and Vezzan [34–36], possibly representing heroic ancestors. As shown by the excavations conducted in Valcamonica and Valtellina in neighboring Lombardy [37], these monuments were erected in megalithic sanctuaries, important meeting places of the alpine Copper Age inhabitants. The existence of long-distance contacts maintained by the local communities, even as far as Central Italy, have been highlighted by the Iceman’s axe made from Tuscan copper [38]. Analogous results obtained for the copper axe from Zug-Riedmatt in Switzerland [39] suggest extended networks and intensive transalpine cultural contacts.

## 2. Methods

The main methodological approach is based on the reconstruction of the technical actions and gestures and their chronological sequence along the entire life cycle of each tool, from raw-material procurement to its final abandonment, its *chaîne opératoire* [40–41]. Analyses took place directly at the South Tyrol Museum of Archaeology in Bozen-Bolzano where the artefacts are preserved. CT was made at Bozen-Bolzano Hospital. All analyses were non-destructive and made without sampling. All necessary permits were obtained for the described study, which complied with all relevant regulations. The permit was given by the Director of the Azienda Musei Provinciali and the Director of the Museum, after the positive response of the Scientific Council of the Museum.

### 2.1 Lithic raw material analysis

The purpose of the study was to describe the chert lithotypes that represent the toolkit with the aim of identifying the geological formations exploited and to delimit possible geographic sources of provenance. The cherts were analyzed under a binocular microscope (Leica Stereomicroscope M205 A) up to 150x equipped with dedicated software for picture management. The chert microfacies were studied in order to recognize the texture and mineralogy, the sedimentary features and structures, the micropalaeontological content and the depositional environment. The colors were classified using Munsell Soil Color Charts. The artefacts were then compared with environmental lithic resources. These have been prospected for and sampled over the years, both in the Southalpine and in the Austroalpine regions from outcrops and secondary deposits, resulting in a large “lithotheque” used for the comparative work, which is almost up-to-date and situated at Ferrara University [42–46]. The approach is to increase knowledge of the regional geology and thus to have a progressively better definition and evaluation of potential local resources. The work is performed by constantly drawing on the earth sciences (geology) and its methods for sampling, mapping and field trip descriptions.

### 2.2 Technological analysis

The technological approach faced the following research questions: Which blanks were used to produce the tools and with which techniques? What is the technical state of the artefacts? What is the knapper’s level of skill? And, finally, is the lithic assemblage homogeneous or not? Pieces were analyzed macroscopically, with optical lenses and by means of macro photos in order to decipher and interpret the scars and their chronology with the aim of recovering the successive intentions of the knapper. Method and terminology are described in the specific literature [47–52]. New drawings of the artefacts were made based on the technological analysis.

### 2.3 Use-wear analysis

The traceological analysis was carried out by means of both the low power approach (LPA) [53–55] and the high power approach (HPA) [56–58]. LPA focuses on the analysis of macro-traces produced by the contact of the lithic tool with the worked material or with a possible handle, in order to interpret the actions carried out and to obtain general information about the hardness of the worked material. HPA is based on the observation of micro-traces allowing a more in-depth interpretation of the worked material. Use-wear was observed at low magnification (20x - 80x) by means of a Hirox KH 7700 3D digital microscope, using a MX-G 5040Z body equipped with an AD-5040Lows and an AD-5040HS lens, and a Leica Stereomicroscope M205 A. Micro-trace analysis was performed by using the fore-mentioned Hirox microscope fitted out with a MXG-10C body and an OL-140II lens (140x- 480x). Both microscopes enable one to obtain fully focused pictures through the overlapping of planes taken at different focus levels [59]. The traces on the archaeological lithic tools were interpreted by means of comparison with traces produced during a dedicated experimental program using replicas of Ötzi’s tools. Furthermore, the experimental reference collection of the research unit “Prehistory and Anthropology” of Siena University was also used. It must be pointed out that analyses were not focused on the study of residues. Therefore, among the residues observed during examination, only the identified ones are reported here.

### 2.4 Computed tomography

A new CT scan was conducted on the hafted tools of the Iceman in order to depict the three-dimensional shape and the structure of the tool portions hidden by the handles and shafts. In addition, the retoucher spike was measured for its radiological density (measured in Hounsfield Units–HU) in order to get information about the material used [60]. Analyses were carried out by a Somatom Definition Flash CT scanner (Siemens, Forchheim, Germany) installed at the Department of Radiology of the Hospital of Bozen-Bolzano. The acquisition parameters were 120 kV, 200 mAs in high resolution technique with slice collimation of 10 x 0.3 mm; images were reconstructed in the axial plane in a soft tissue (U30u) and a bone tissue algorithm (U90u) with a slice thickness of 0.4 mm (overlapping of 0.2 mm). A second acquisition was performed using the Dual Energy (DE) technique with the following exposure parameters: 80/140 kV and 250/97 mAs with a slice collimation of 32 x 0.6 mm followed by image reconstruction of 0.6 mm (overlapping 0.4 mm) in a soft tissue Kernel (B30s). Post-processing of the images included multiplanar reformations in the coronal and sagittal plane and volume rendering techniques (VRT).

### 2.5 Typological comparison

The study focused on the comparison of the dagger, the three arrowheads and the endscraper with more or less contemporary artefacts of the Alpine and perialpine Copper Age contexts represented in the literature. The publications consulted were restricted to the Northern Italian Alpine regions, as well as Swiss, Austrian, and southern German territories. Only the artefacts depicted were taken into consideration. The aim was to gain information about the cultural background of the Iceman. The ^14^C datings reported in the text have been recalibrated with Oxcal ORAU using the IntCal 13 curve within 2σ confidence range.

## 3. Technology and use-wear

### 3.1 Dagger

**Fig 1:**
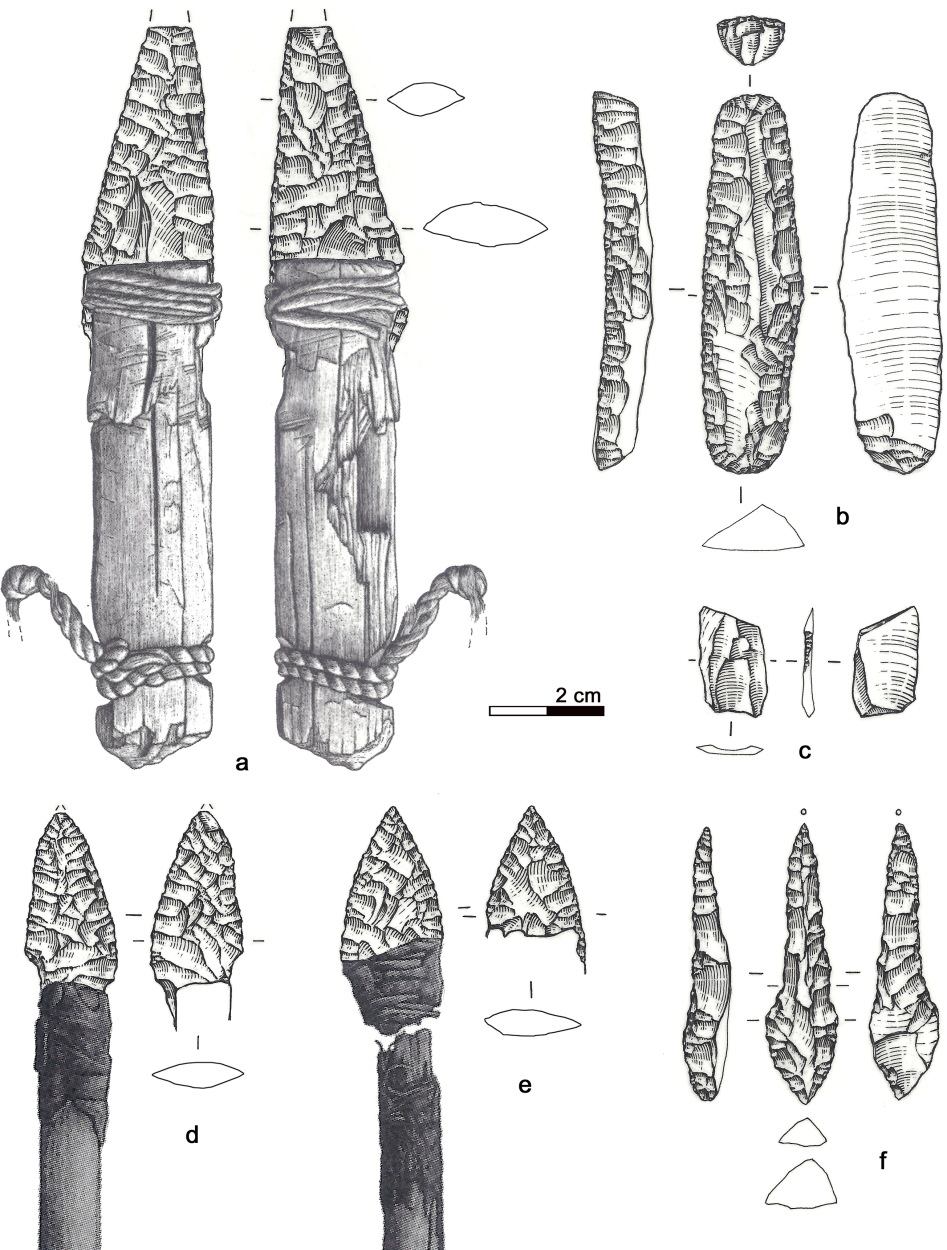
The Iceman lithic assemblage. a) Dagger, b) Endscraper, c) Borer, d) Arrowhead 14, e) Arrowhead 12, f) Small flake.

The 132 mm long dagger comprises a chert blade hafted on a handle of ash wood (Fig 1A) [9]. The blade (69 x 24 x 7.7 mm) is inserted into a deep slit and held in place with sinews, without any tar. A basal groove on the flat handle hosts a string made from lime-tree fibres which served to extract the dagger from its bast scabbard.

**Fig 2:**
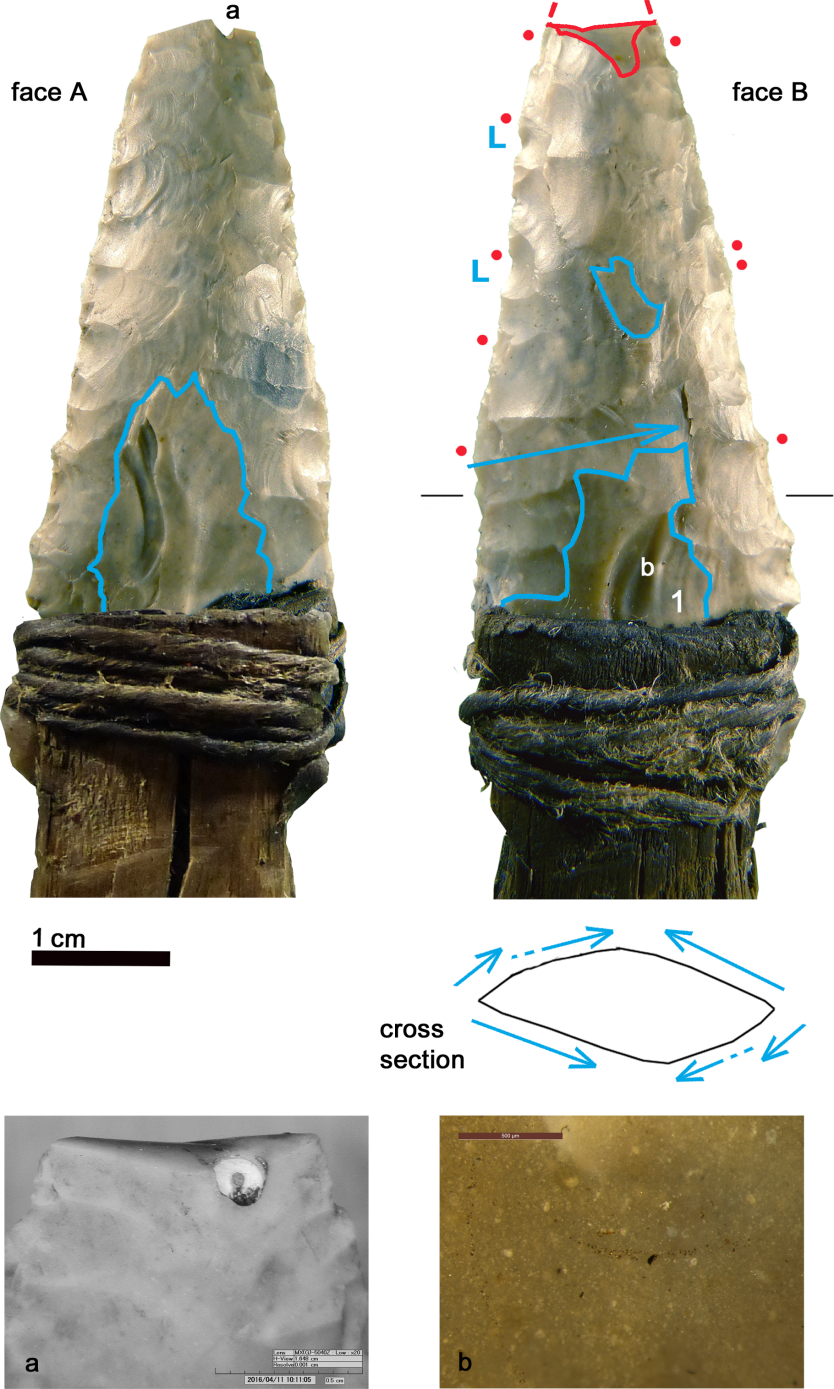
Dagger. Red outline = ancient fracture; Blue outline = remnants of preform; blue arrow = long detachment; red dots = location of use-wear; 1 = wide hinged scar; L = last crushed detachments; a = fracture of the tip, corresponding to an embedded fossil; b = Scratch on the dagger surface produced by a copper object.

The dagger blade, with biconvex cross section and straight profile, originates from a bifacial preform rather than from a blade. In fact, the possible remnants of the preform are identified on both faces (Fig 2, blue outline). The width of the hinged scar on face B (Fig 2, 1) indicates that thinning was done with direct percussion and not with pressure. The subsequent retouch, bilateral and bifacial, was clearly made with pressure: the negatives show the characteristic regular obliquity and rise up slightly. Some scars are very long and overpass the mid-line of the dagger. Several characteristics indicate that the tool had previously been resharpened: a) the asymmetrical cross section, created by at least 2 series of pressure retouches from one edge; b) the fact that most pressure removals meet at the mid-line shows that the previous cross section was already regularly biconvex, i.e. that it was already shaped by pressure retouching. A reduction of the dagger size by resharpenings is therefore confirmed (see p. 126 in [9]). The latest attempt at resharpening is evidenced by short removals which are crushed or have opposed obliquity (Fig 2L). The knapper who made the dagger had a medium to good skill in pressure flaking, detaching regular, oblique and uprising negatives. Only the final attempt at resharpening appears of lower quality.

**Fig 3:**
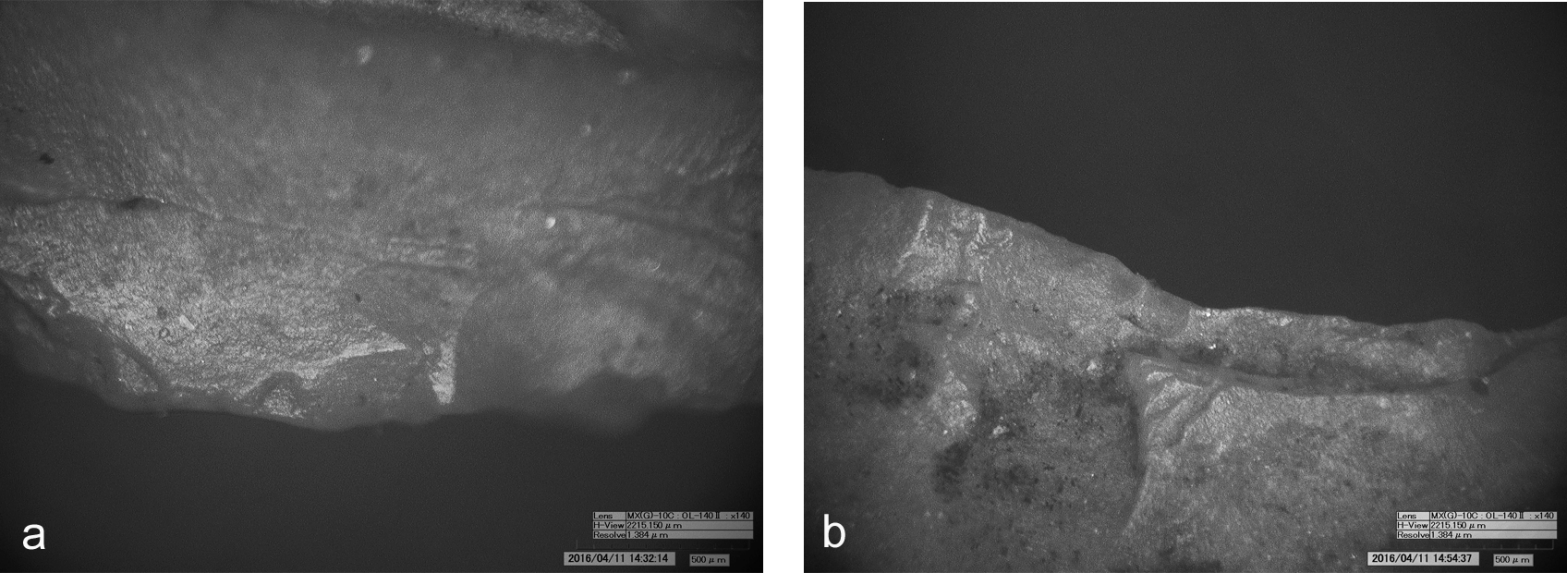
Use-wear on dagger. a) Use-wear polish referable to working soft animal material (140x); b) Use-wear polish compatible with the contact with the bast scabbard (140x).

The dagger tip has an ancient feather terminating bending fracture corresponding to an unsilicified calcitic crinoid which had weakened the chert (see chapter 4.6) (Fig 2A). The fracture could have happened during use [61]. Besides this, the blade bears only few microscopic wear traces: a light edge rounding, few micro-scars and few polishes organized in spots only along the edges (Fig 2, red dots). Polishes are not well developed. Some are consistent with contact with the bast scabbard, others relate to the cutting of soft animal material, either meat or hide (Fig 3A and 3B). The latter observation is in line with preceding analyses (see p. 128 in [9]). All-in-all the wear suggests that the dagger blade, at least in its actual state after resharpening, did not have an intensive and prolonged use. During raw material analysis copper traces have been detected on the blade surface (Fig 2B). The two scratches seem to have been provoked by accidental contact with a copper artefact, for example with Ötzi‘s axe. It is not clear whether the scratches are ancient, or whether they arose as a result of the recovery operation.

### 3.2 Arrowhead of arrow 12

Arrow 12, formed by a shaft with feather fletching combined with a foreshaft, is armed with a 46 x 16.3 x 4.7 mm sized lithic arrowhead. It had been inserted into a split, fixed with vegetal thread and then covered with birch-bark tar (Fig 1D). The x-ray images of the full quiver show the fragmentation of this arrowhead, found with the tang fragment still inserted in the foreshaft and the distal fragment lying higher up [9]. The pieces were glued together during restoration.

**Fig 4:**
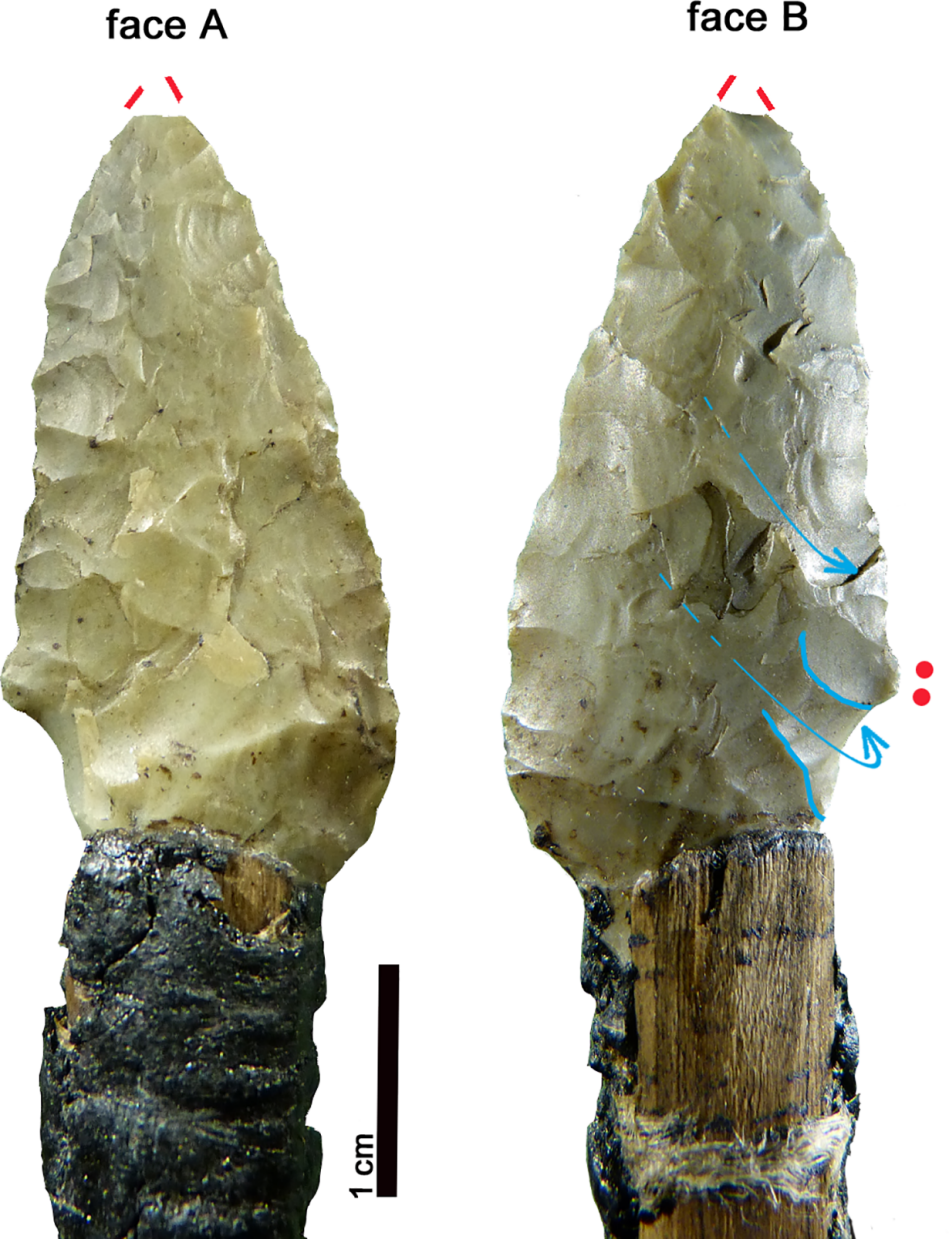
Arrowhead 12. blue arrow = plunged scar; red dots = location of use-wear.

**Fig 5:**
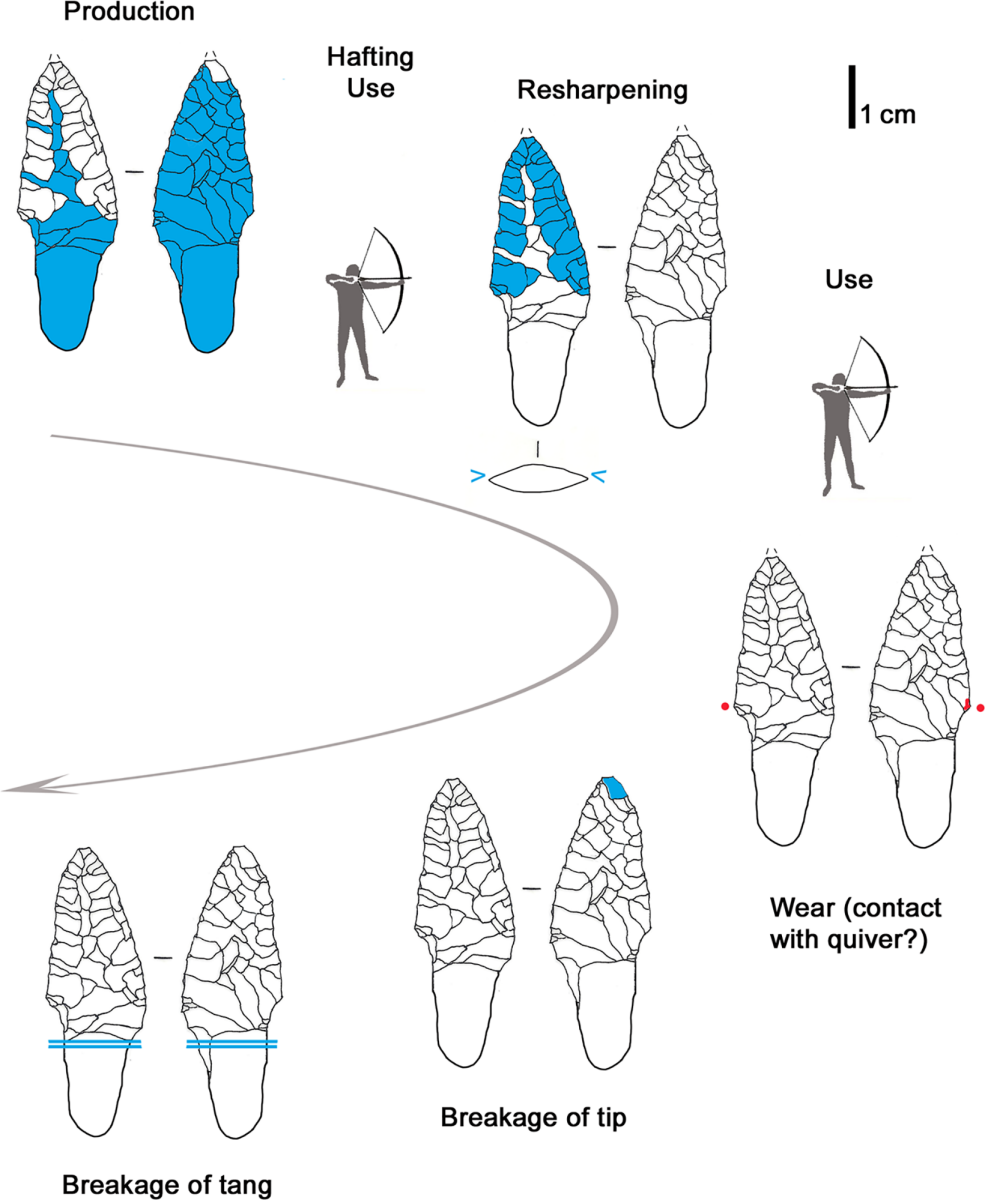
Arrowhead 12: Sequence of actions.

To produce arrowhead 12 a regular, well selected flake blank was used, as shown by the fairly biconvex and symmetrical cross section of the projectile. The bifacial bilateral retouch is made with pressure. Some thinning scars are very invasive: a long detachment reached as far as the opposite edge and damaged it by plunging, which provoked the basal notch (Fig 4). The sequence of the detachments does not show much organization and lacks any systematic arrangement. Nevertheless the knapper did a gradual and careful perfectioning of outline and profile, achieving very thin and sharp edges. The piece has been resharpened at least once as shown by the superimposition of retouches on face A (Fig 5). The arrowhead appears to be the work of a knapper with a good skill in pressure retouching, who was able to carefully modify the piece bit by bit. The achievement of a good result is testified by the sharp edges without crushings.

**Fig 6:**
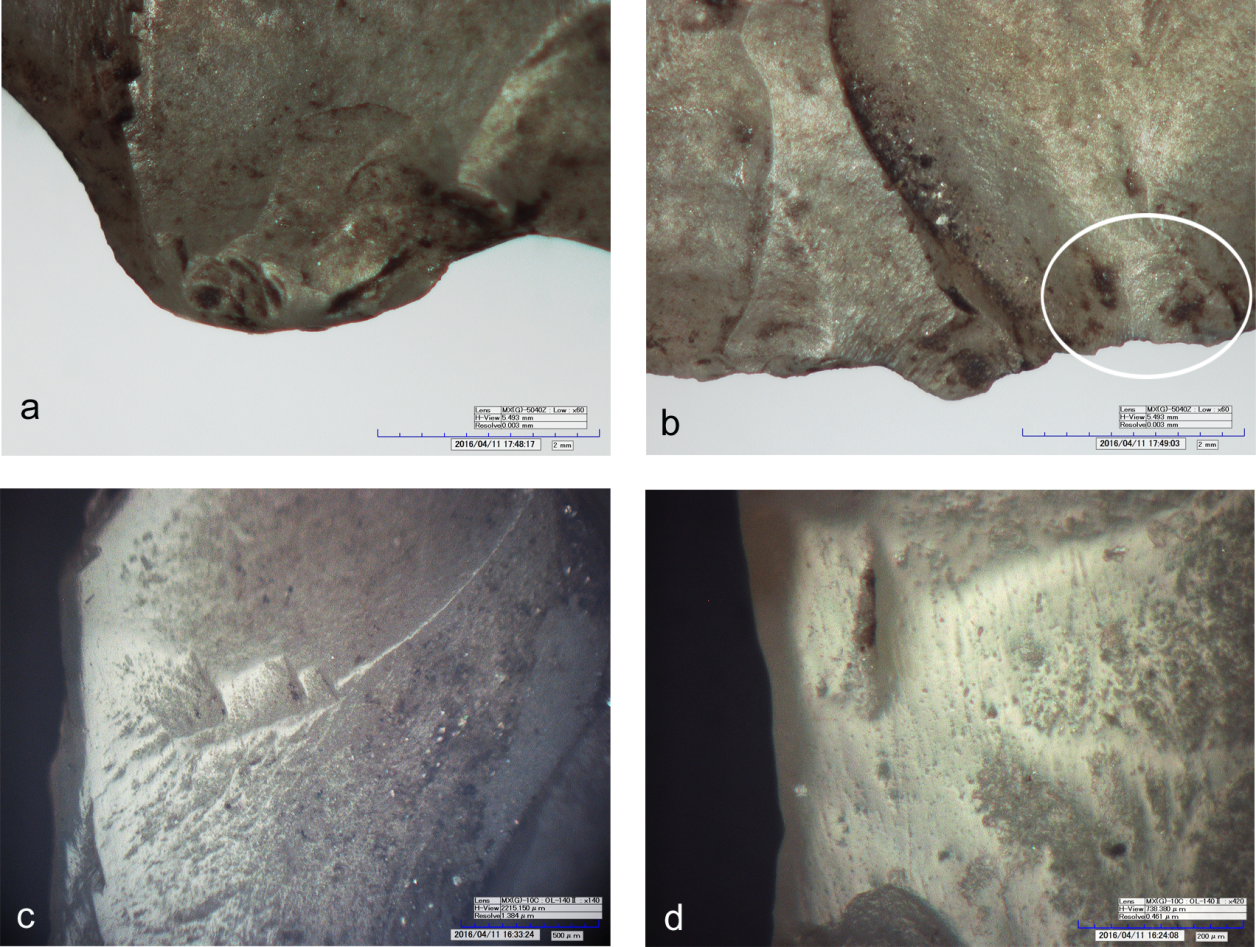
Use-wear on arrowhead 12. a) Edge rounding localized on the maximum width of the arrowhead (60x); b) Dark residues, probably birch-bark-tar, on the maximum width of the point (60x). **Use-wear on arrowhead 14**: c) Well developed luster and longitudinal striations from cutting siliceous plants (420x); d) Micropolish and longitudinal striations from cutting siliceous plants (420x).

The fracture of the tip occurred during Ötzi’s time. It is not a diagnostic impact fracture as stated by Loy [9, 62]. But a breakage during retouching cannot be considered either, as in such a case the knapper would have repointed the tip. The arrow point does not show any wear from use, so that earlier suggestions cannot be confirmed (“cutting of hide” [9, 62]). The only microscopic trace is a slight and not extended edge rounding localized above the lateral notch (Figs 4 and 6A). Its origin may be from the contact with the leather of the quiver. An arrowhead with a comparable alteration was found at the Copper Age burial site of Lunghezzina near Rome [63]. Both faces of the piece are covered by numerous tiny tar drops (Fig 6B).

### 3.3 Arrowhead of arrow 14

The pointed extremity of arrow 14, formed by a lithic arrowhead fixed with thread and birch-bark-tar, lay severed from its *viburnum* shaft inside the quiver (see supplement 6 in [9]) (Fig 1E). This breakage also affected the base of the lithic tang, which shows a bending fracture. The tang fragment has not been found.

**Fig 7:**
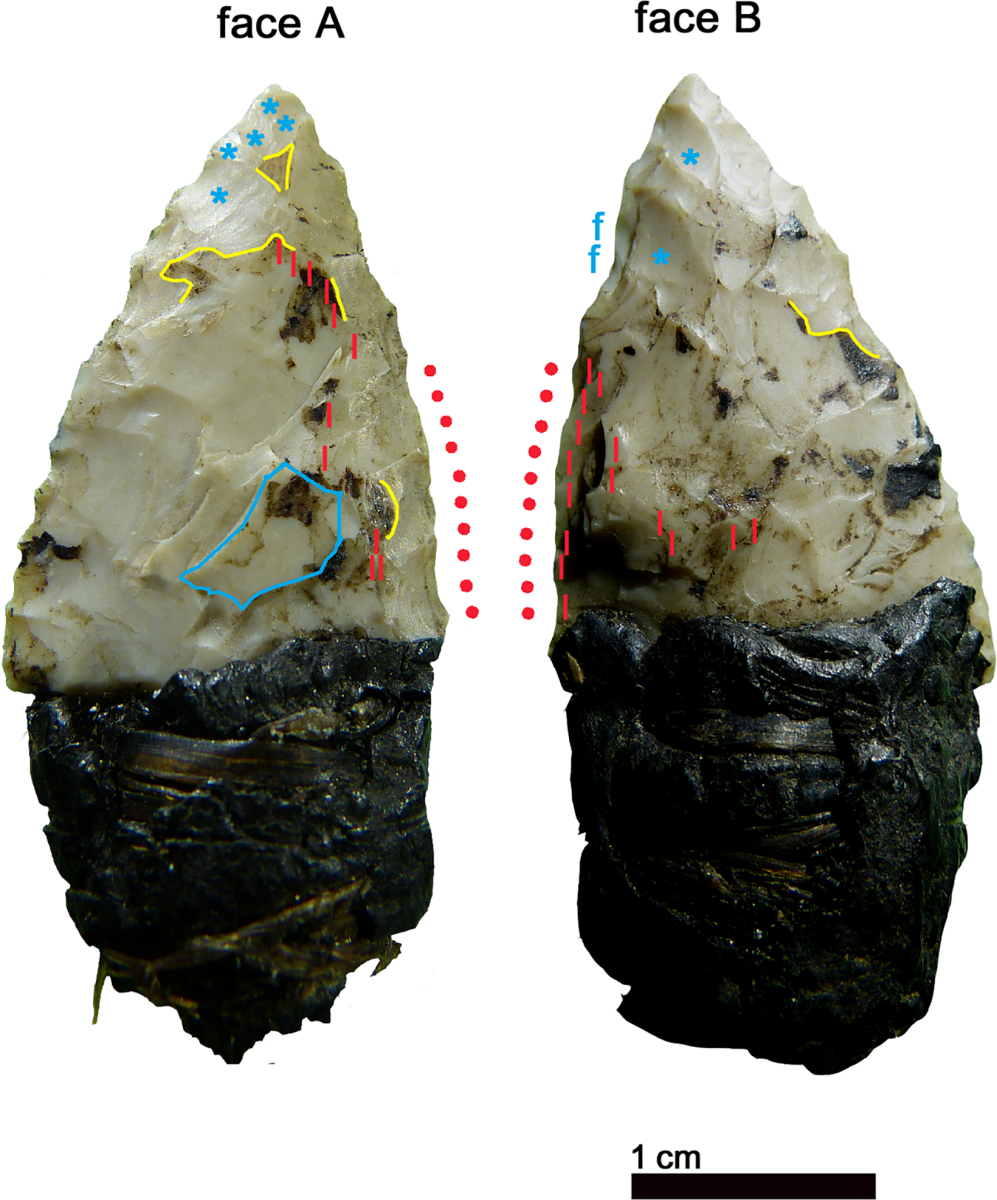
Arrowhead 14. blue outline = remnant of flake blank; yellow line = birch-bark-tar interrupted by later detachments; red dots = location of gloss; red lines = gloss visible to the naked eye; blue stars = last and clean detachments; f = fresh detachments (recent?).

**Fig 8:**
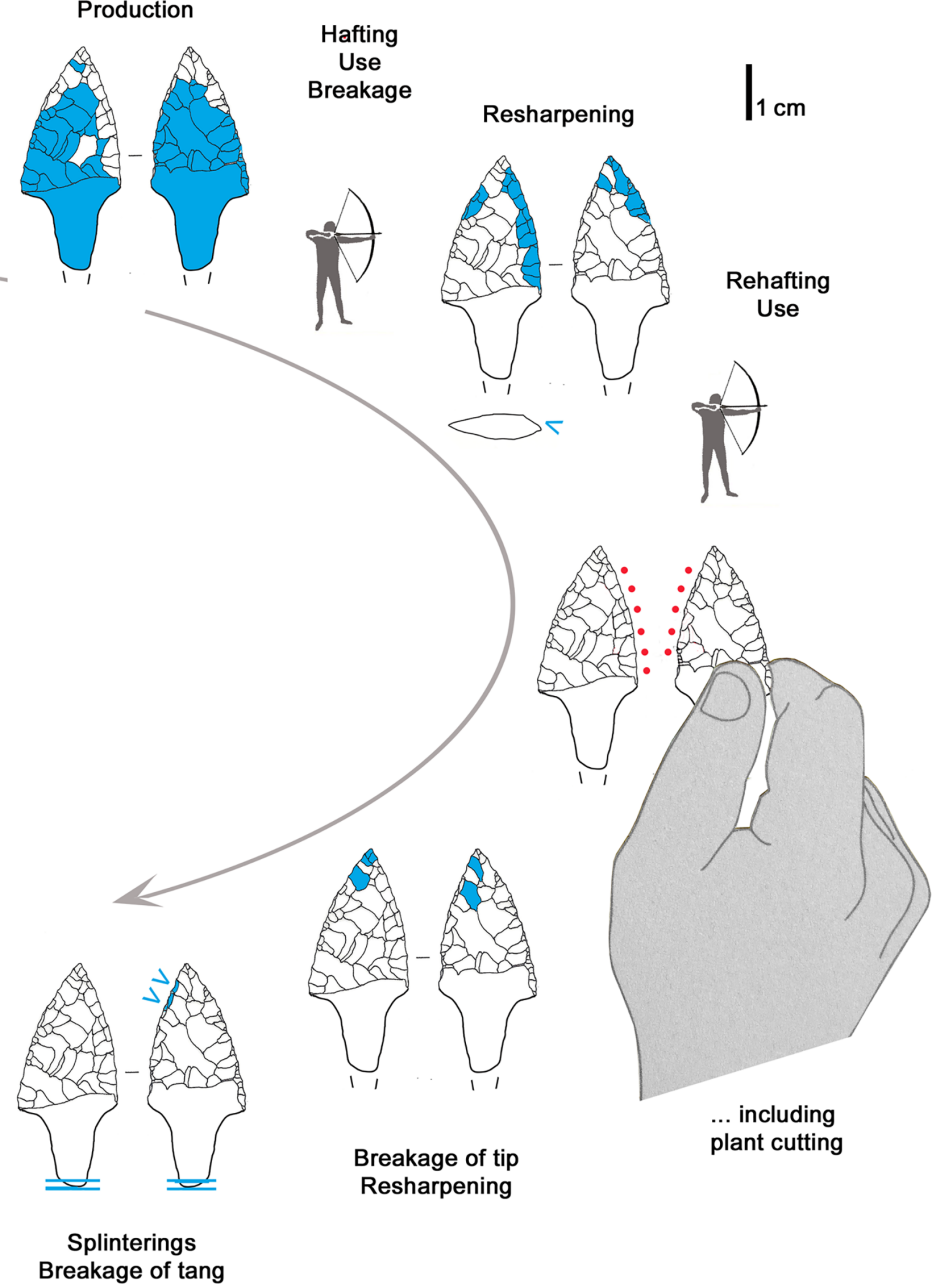
Arrowhead 14: Sequence of actions.

The production of the 39 x 19 x 4.5 mm sized arrowhead started from a flake blank, as shown by a dorsal scar preserved on face A (Fig 7, blue outline). Both faces were shaped with flat retouch made by pressure, with some long scars reaching the opposite edge. Judging from the superimposition of scars and tar traces, the piece underwent at least two resharpenings before reaching the final state (Figs 7 and 8). Such repairs determined the reduced size and the asymmetric cross section and outline. The pressure retouch, whose style is similar to arrowhead 12, reveals a rather fine and careful working, with few crushings, giving very thin and sharp edges. The arrowhead tip is intact and particularly clean. The arrowhead is covered with abundant tar residues. There are also a few hair fragments embedded in pitch as indicated by Loy [64]. As already observed [9, 64] the item displays an evident gloss from the cutting of siliceous plants. This thoroughly pervades face B and is limited to the high points of face A (Fig 7, red lines). The edge itself appears very rounded, showing that its use occurred after the resharpening. The hypothesis that Ötzi made this arrowhead by recycling a former sickle blade must therefore be rejected [64]. The polished surface is flat and glossy (Fig 6C and 6D). Micropits have a filled-in appearance. Striations are not numerous, and are narrow and parallel to the active edge.

The sequence of different actions and modifications reconstructed from the superimposition of retouch, tar and gloss evidences the long history of the projectile point (Fig 8), with repeated repair, rehafting and use, intermitted by “improper”, maybe expedient, use for plant cutting. A short time before his death Ötzi resharpened the tip, without having then occasion to use it again.

### 3.4 Endscraper

**Fig 9:**
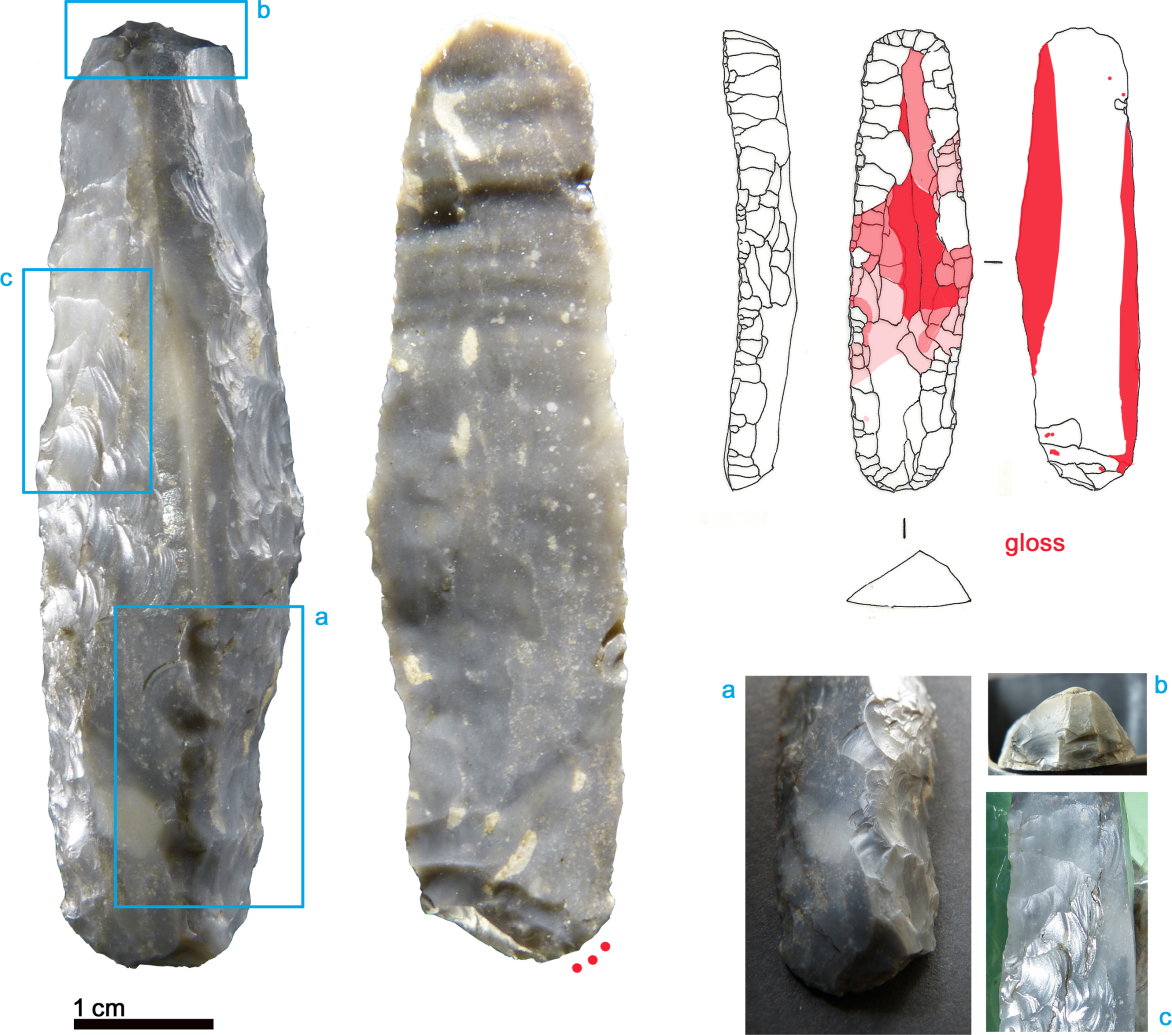
Endscraper. a) overstepping negatives made with pressure; b) endscraper front made with direct percussion; c) superimposition of fresh on glossy negatives. Upper right: distribution and intensity of gloss; red dots = location of small crushings.

The largest lithic tool carried in the belt poach, the 66.5 x 18.2 x 8.3 mm sized endscraper (Fig 1B), is made from a thick blade with triangular cross section, whose original size was 23 mm wide and at least 100–110 mm long considering its profile. Considering that such thickness and width are difficult to achieve by pressure, one can suppose that the blade was most probably detached with indirect percussion. According to the dorsal negatives it was part of a unidirectional blade production sequence. The lateral edges show several series of retouch (Fig 9C). This bilateral resharpening as well as the flattening of the bulb are made with pressure. Characteristic features are the neat negatives which overstep the central arris (*outrepassages chevauchants* [50]) (Fig 9A). Only the endscraper front, being steep and displaying short hinged negatives, could have been realized by direct percussion (Fig 9B). The tool is in a highly advanced technical state; hardly any further reduction would have been possible. The pressure retouch reveals a medium to good level of skill of the knapper, whereas the production of the original blade by indirect percussion was surely done by an expert knapper.

**Fig 10:**
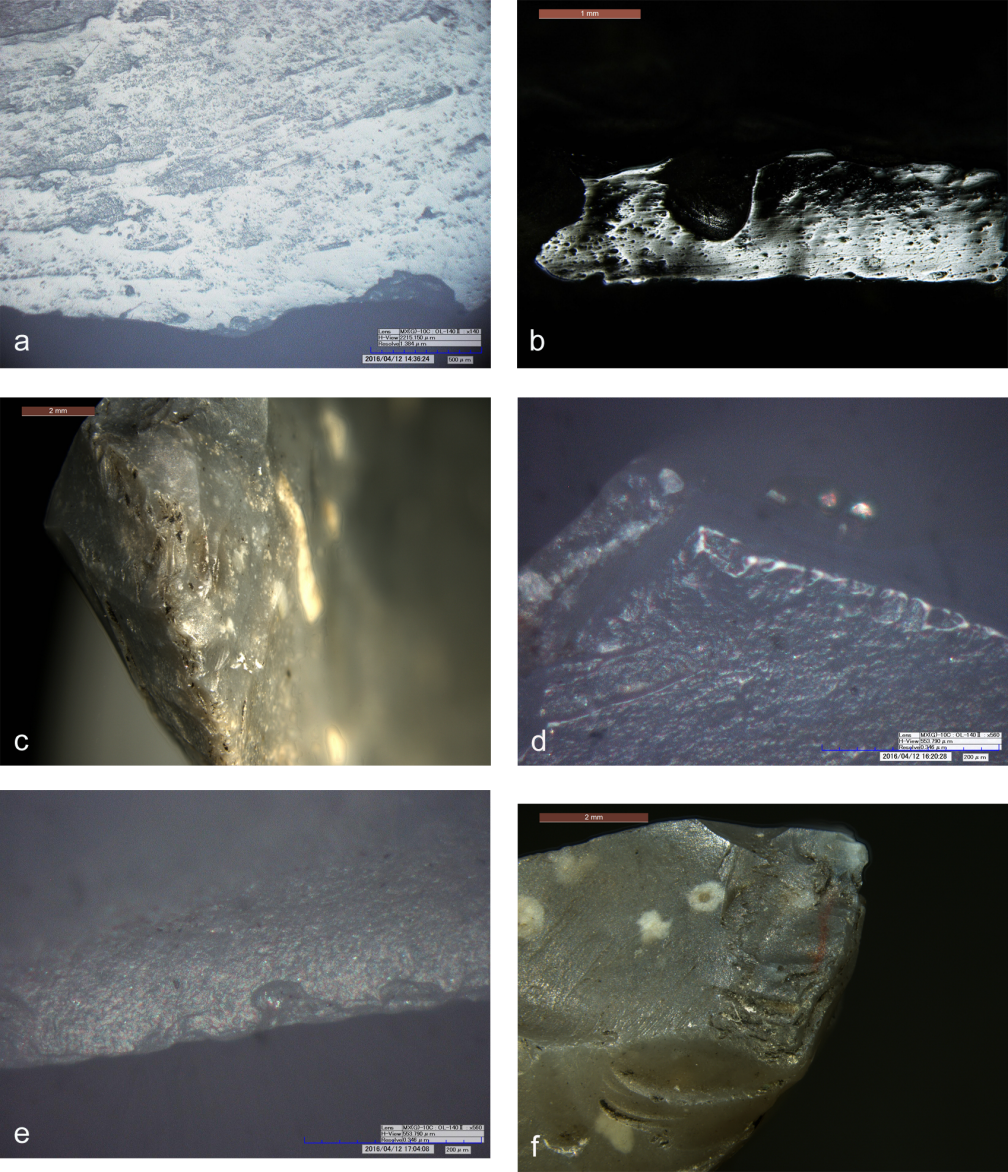
Use-wear on endscraper. a) Very developed luster from cutting siliceous plants on the ventral face (140x); b) Very developed gloss and longitudinal striations on the dorsal face; c) Crushing and smoothed areas consistent with the use as “strike-a-light”. **Use-wear on borer.** d) Micropolishing on the tip referable to contact with hard material (560x); e) Micropolishing on a lateral edge referable to transport or prehension (560x); f) Light crushing referable to a use as a “strike-a-light”.

The tool edges show a very developed use-wear, a strong gloss, observable on both faces (Fig 9, upper right). The variable intensity of the gloss is consistent with a prolonged cutting action that continued over time, intermitted by edge resharpenings. At microscopic observation the polished surface is smooth and glossy (Fig 10A and 10B). There are also many micropits, some of them comet-shaped with a filled-in appearance. Striations are not abundant. They are narrow and run parallel to the active lateral edges, which are both very rounded. The traces were produced by cutting siliceous plants as already stated in previous studies [9, 61]. The knife was evidently used without handle. The varying pervasiveness of the gloss, from marginal to very deep, derives from handling the tool with a very low angle during the cutting action. Loy’s statement about the use of the central arris as an active edge can be rejected: the edge itself does not bear wear traces [9, 62].

**Fig 11:**
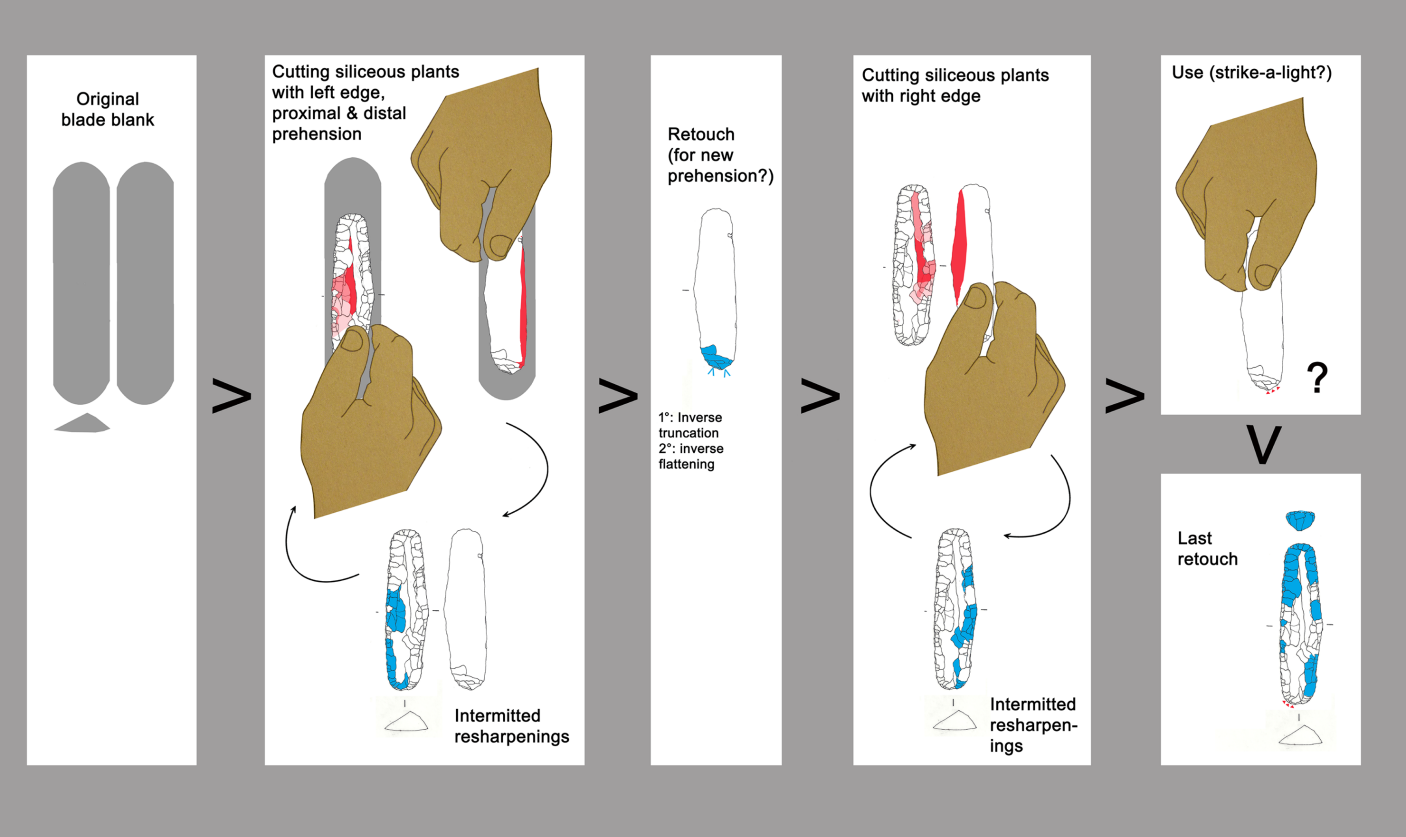
Endscraper: Sequence of actions.

The spatial distribution of the gloss combined with the stratigraphy of retouch allows us to propose a possible sequence of gestures, showing changing prehension modes for using the left and the right lateral edge (Fig 11). The person who used the cutting tool was a right-hander. Interestingly Ötzi never used the tool after its last modification into an endscraper as it does not show any traces of having been used. The opposite proximal end instead displays edge rounding, edge removal and crushing observable both macro- and microscopically. These traces, although not very developed, have an aspect suggesting use as a “strike-a-light” [65, 66] (Fig 10C). An expedient use for such a task can therefore be taken into consideration, as already hypothesized by Chelidonio [67].

### 3.5 Borer

**Fig 12:**
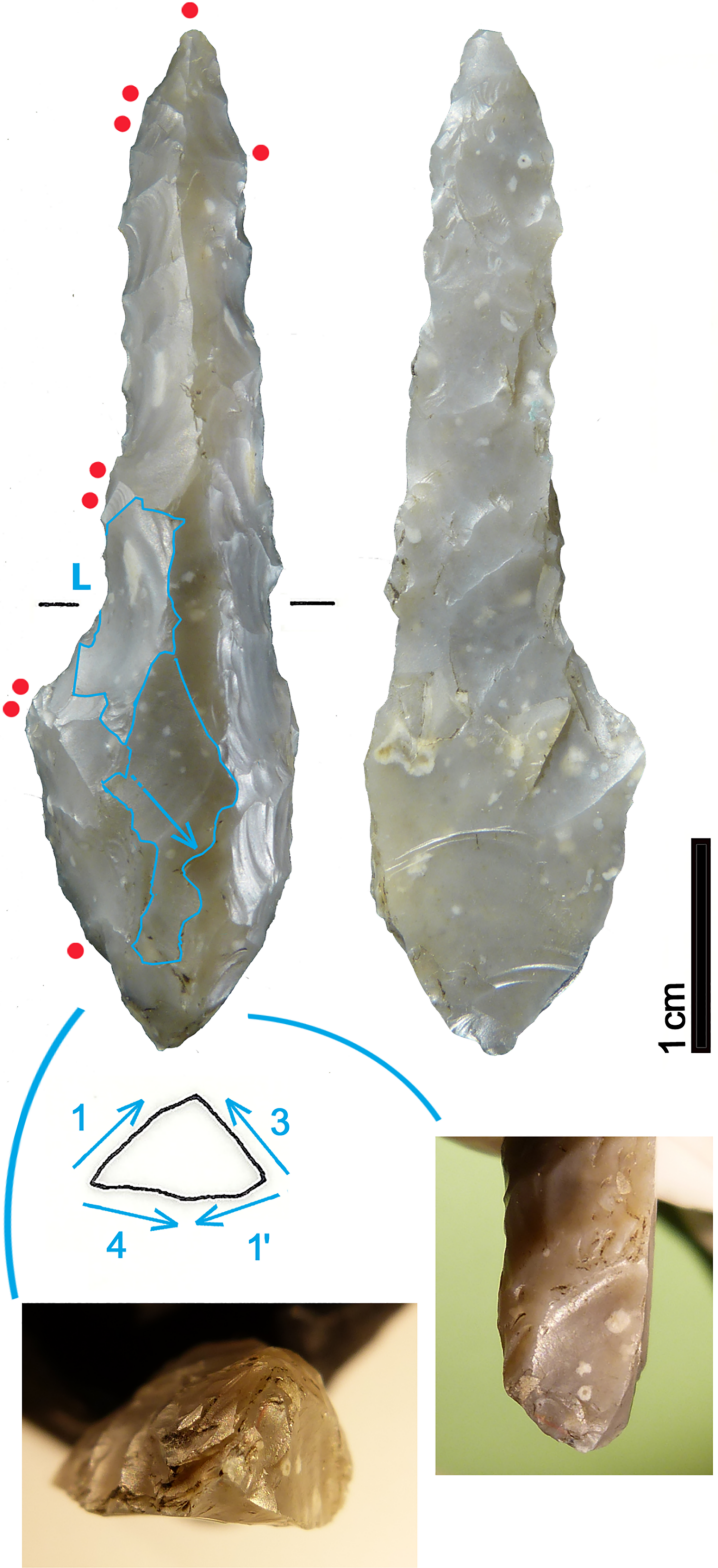
Borer. blue arrow = oblique dorsal negative; L = Last detachment; red dots = location of use-wear; cross section with sequence of retouch; lower right left and right: details of crushings.

The 48.5 mm long artefact known as a borer has its maximum thickness at its maximum width (7.8 x 13.5 mm) (Fig 1F). The original blank is identified as a rather thick blade from the maintenance of a blade core: the oblique dorsal negative is consistent with an under-crest blade (*lame sous-crête*) from a crest placed on the core back (Fig 12, blue arrow). The blade was probably detached by indirect percussion rather than by pressure. The series of retouches, at least two, were made by pressure. Also the last, 9 mm wide negative (Fig 12L), was clearly made by pressure because it is detached from a concavity. Such a detachment, which needs a lot of force, shows the intention of building the long and narrow extremity, shaped by bifacial bilateral retouching (Fig 12, cross section). The borer is in an advanced technical state: no further transformations would have been possible. Regarding knapping skill the piece shows a correct technique of blade detachment. Also the pressure retouch is executed with correct and effective skill.

Already by a macroscopic examination one notes the evident difference between the “freshly” retouched distal half, of opaque appearance, and the proximal part which has a “worn” aspect due to a more reflecting surface and dark residues in the depressions (Fig 12). The borer does not show evident traces of utilization, which contradicts previous information (“wood and bone working”) [9, 62]. On the pointed extremity only a few spots of randomly localized wear are detected (Fig 10D). These are too tiny and do not seem to have been produced by an activity, but rather by contact with a hard material, possibly during transport in the belt pouch. A few other spots of polishing on the edges could also be related to transport or to prehension (Fig 10E). On the proximal end of the artefact, corresponding to a normally oriented lateral detachment, some traces of crushing are visible (Fig 12, low; Fig 10F). Even though less developed, they seem very similar to those detected on the endscraper, probably related to its use as a strike-a-light. A sample taken from this area contained “dark brown to black particulate material which eventually settled to the bottom of the sample tube” [62]. The question arises, whether these particles were pyrite or marcasite.

**Fig 13:**
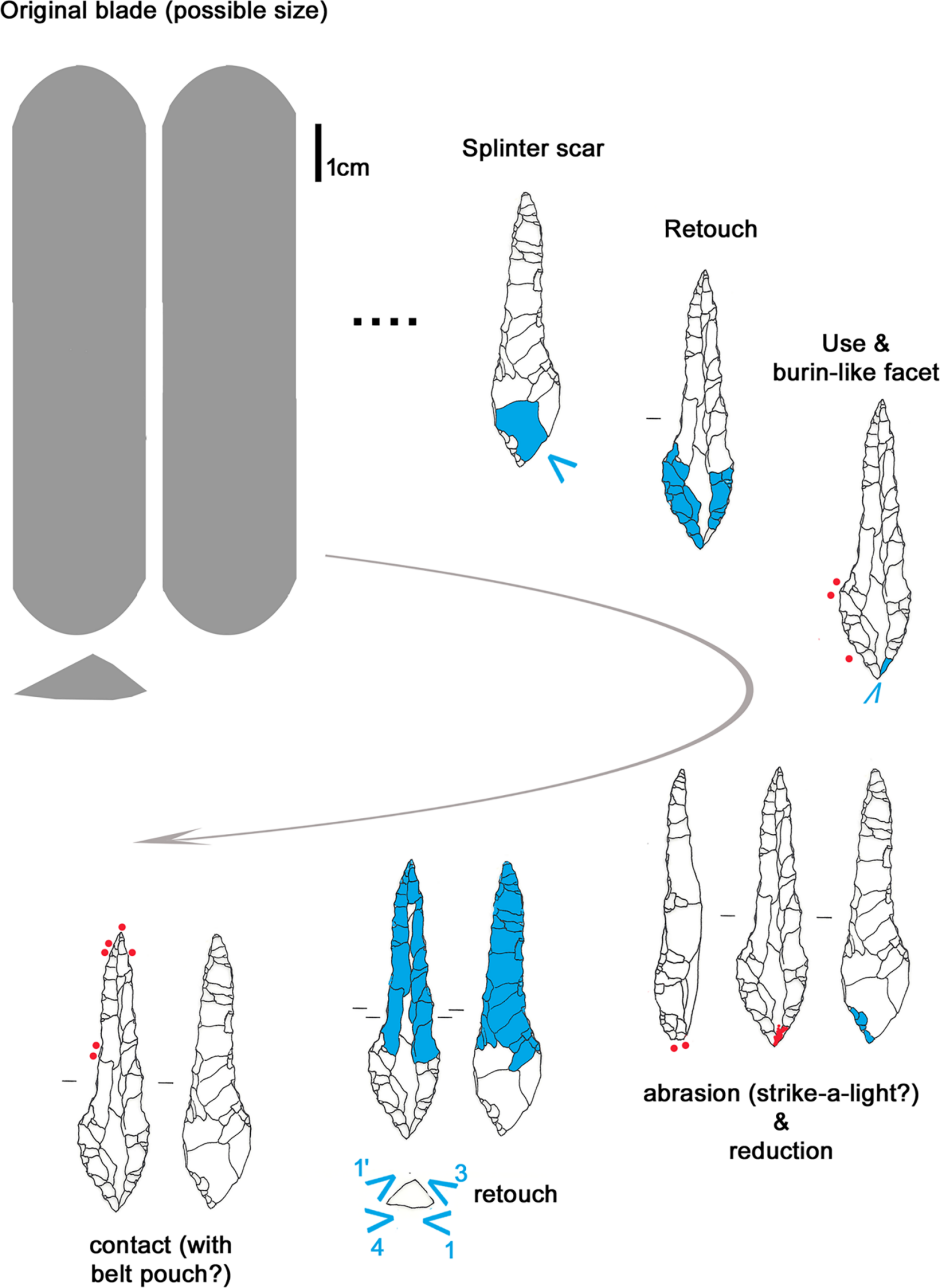
Borer: Sequence of actions.

The long tool-history shows alternating modifications and use (Fig 13). As with the endscraper, the last retouch, the shaping of the robust pointed extremity, was not followed by the use it had been intended for.

### 3.6 Small flake

**Fig 14:**
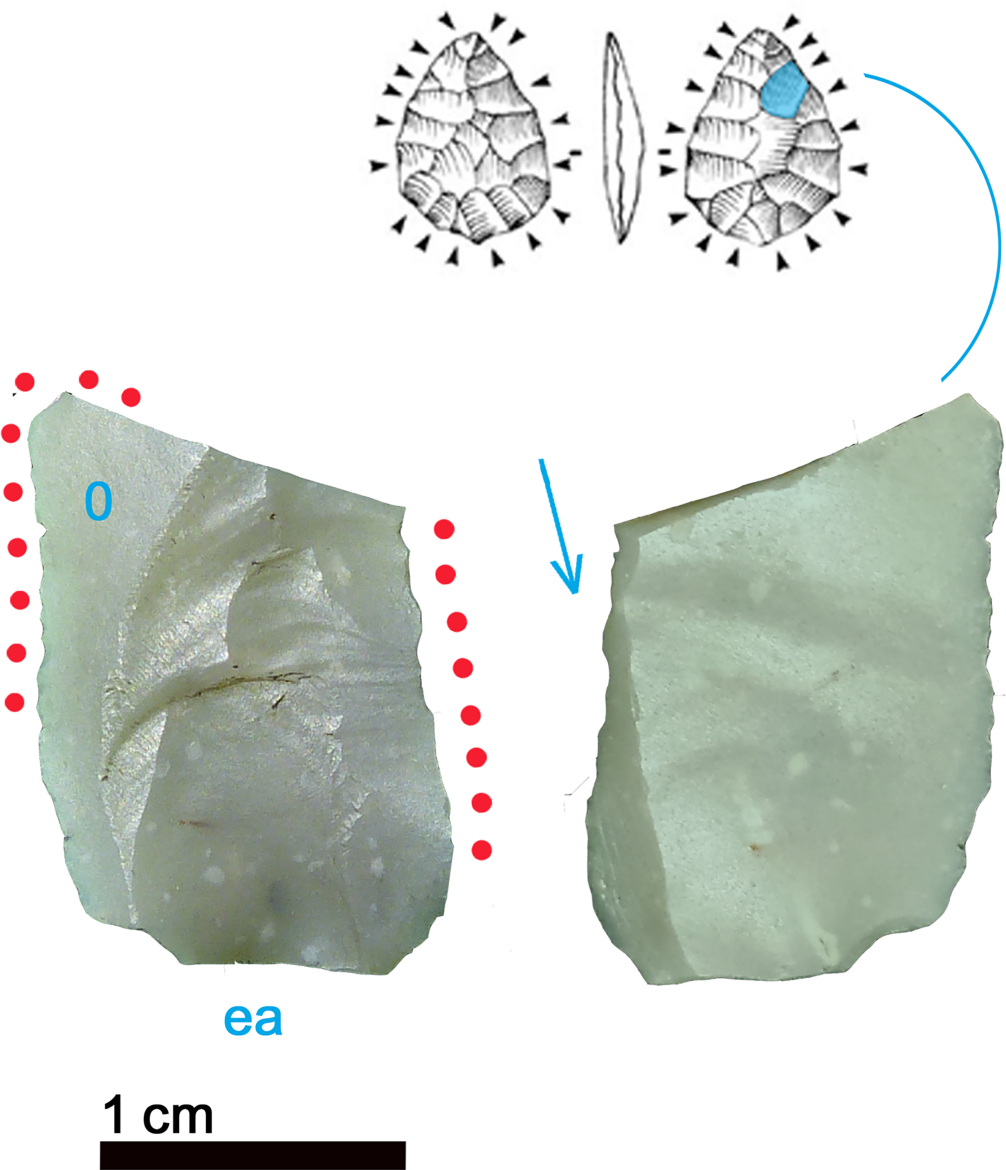
Small flake. 0 = remnant of ventral face of flake blank; ea = spontaneous negative; arrow = burin-like accident; red dots = location of use-wear.

The smallest item contained in the belt poach is a small flake of 19 x 12.5 x 1.6 mm size (Fig 1C). Its origin is revealed by a flat dorsal facet interpreted as a remnant of the ventral face of a flake blank (Fig 14, 0). The piece, a thinning flake, was detached during an early moment of thinning aimed at transforming a flake blank into a bifacial preform for a dagger or arrowhead. The dihedral striking platform, the small lip and the absence of any impact mark indicate that it was detached by direct organic percussion [68]. The moment of detachment provoked a flaking accident, a so called *“epiphenomène anterieur”* in the form of a spontaneous negative (Fig 14, ea). A further accident caused the transversal and lateral fracture of the flake: in fact, when the percussion shock comes to stop against an obstacle (e.g. hinging, previous step fracture) and breaks the flake, an additional lateral fracture running backwards towards the striking platform can occur. Such “burin-like” accident (*pseudo-coup de burin*) is visible on the ventral face (Fig 14, arrow). This means that Ötzi collected the piece already as a fragment. Such thinning flakes, which are waste material, would be found in abundance at a specialist knapper’s workshop.

**Fig 15:**
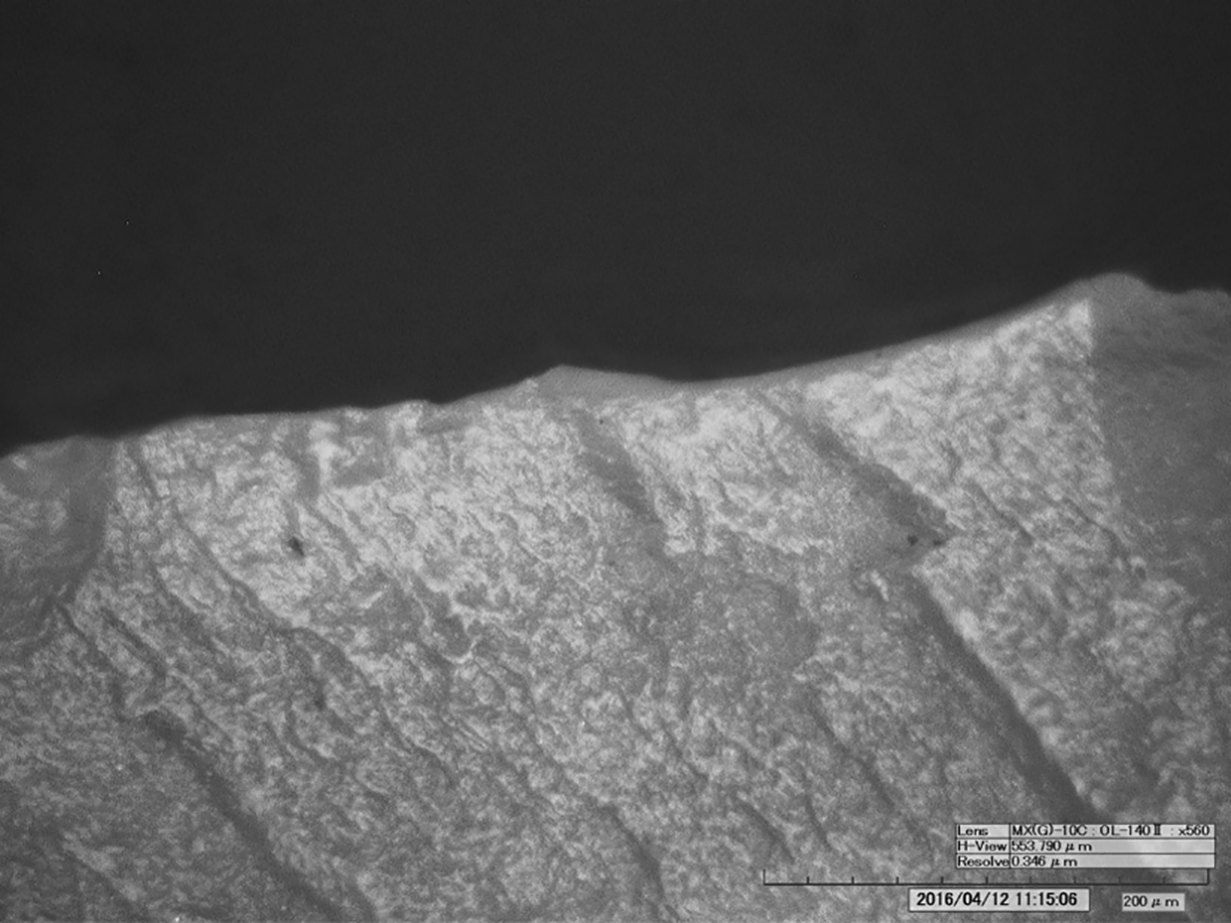
Use-wear on small flake. Use-wear polish from cutting vegetal material (soft wood or reeds) (560x).

The flake bears evident traces of use, as already stated by other authors [9, 62]. The very sharp lateral edges, as well as the distal fracture facet, show scars and polishes that are consistent with a cutting action of vegetal material, in particular soft wood or reeds (Figs 14 and 15). No identifiable residues were detected on the artefact, not even the described feather barbule [62].

### 3.7 Retoucher or pressure flaker

**Fig 16:**
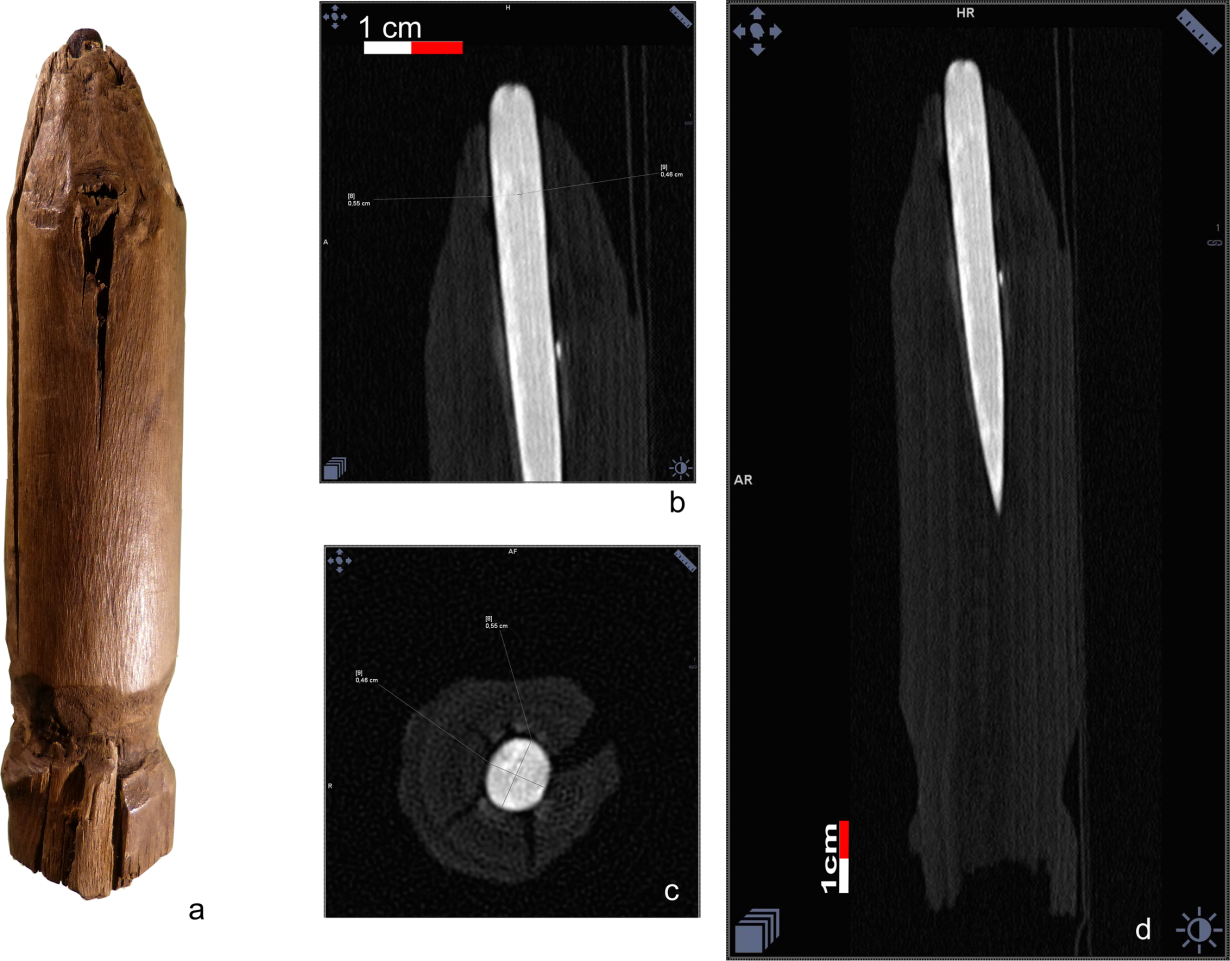
Retoucher. a) the wooden handle and antler tip, b-c) detail of the radiological structure of the antler spike, displaying the cortical and the cancellous parts, d) CT image depicting its radiological structure.

**Fig 17:**
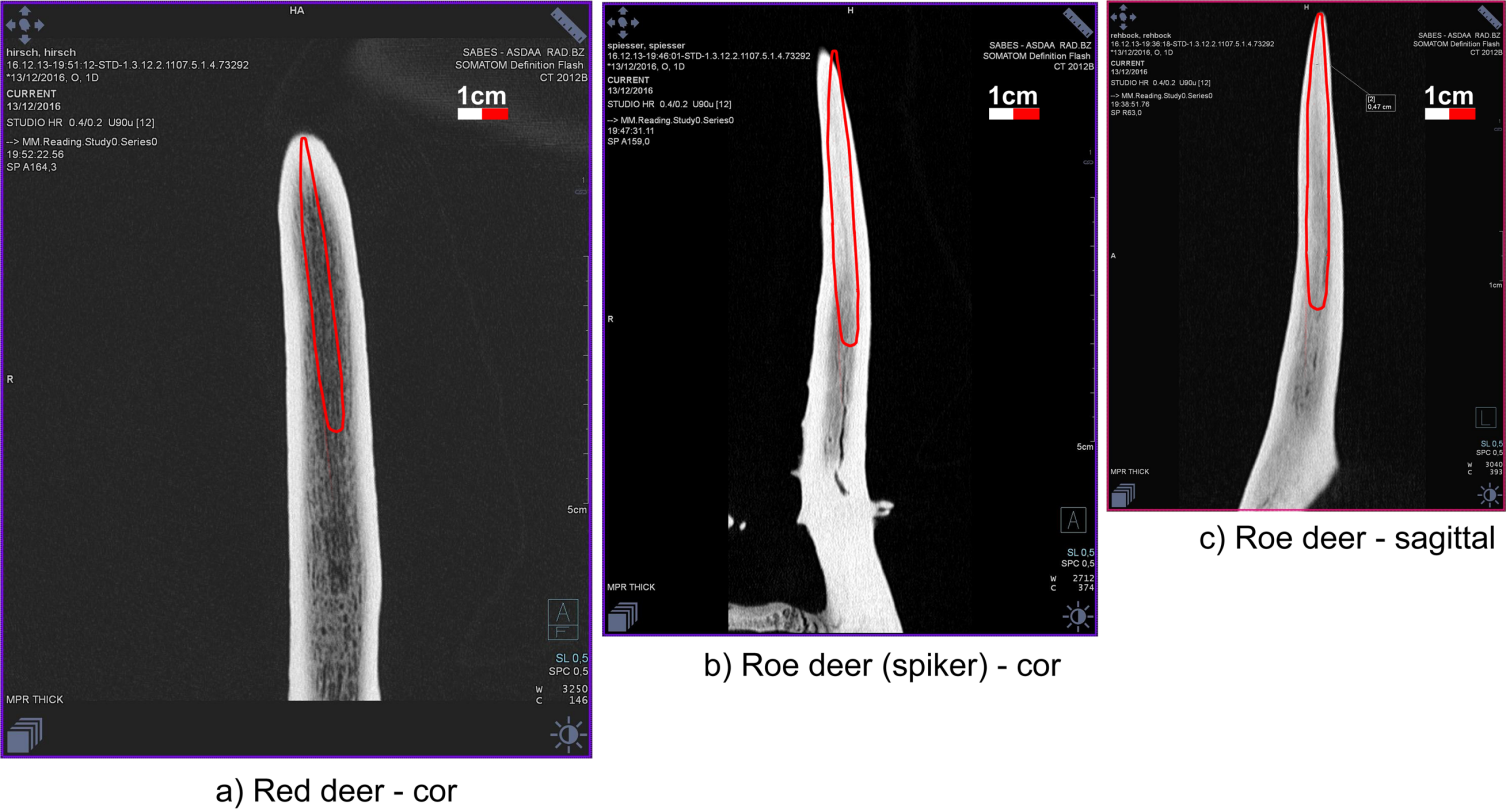
Shape and radiological structure of natural antler points. a) red deer, b) roe deer (yearling), c) adult roe deer. Size comparison with the antler spike of Ötzi’s retoucher (red outline).

Ötzi’s retoucher, a pressure flaker, is formed by an antler spike, deeply inserted into the medullary canal of a stripped branch of lime-tree [9, 16, 69–70]. The upper extremity of the wooden handle is trimmed, the lower cut straight. A basal groove served presumably to hold a cord (Fig 16A and 16B). The 60 mm long antler spike has a pointed base, whereas the tip, projecting for about 4 mm, is blunt. The CT image shows its radiological structure (Fig 16C and 16D): an inner cancellous portion surrounded by a more compact cortical one, which is absent only at the upper tool-end. This signifies that the antler spike is not made from *spongiosa* as hypothesized [70], but rather from a natural straight antler point. Due to the limited thickness, 55 x 45 mm at most, a red deer point is highly improbable as it has a much wider cancellous antler portion (Fig 17A). A strong morphological similarity exits with antler points of the roe deer, the only other antler-bearing species locally available in Ötzi‘s time (Fig 17B and 17C), as shown by the results of three experiments which were carried out.

The thin cortex could be the result of a superficial shaping of the roe deer beam. The comparison of the radiological density of the cortical and cancellous parts confirms the similarity with roe deer, whereas red deer has a much lower cancellous density (Table 1).

**Table 1:** CT measurements of the radiological density of the retoucher spike antler point samples.

	Retoucher spike	Roe deer antler points	Red deer antler point
Antler portion	HU	HU	HU
Cortical	1600–1700	1550–1600	1400
Cancellous	1250	1460	- 85

Mean values expressed in Hounsfield Units (HU).

**Fig 18:**
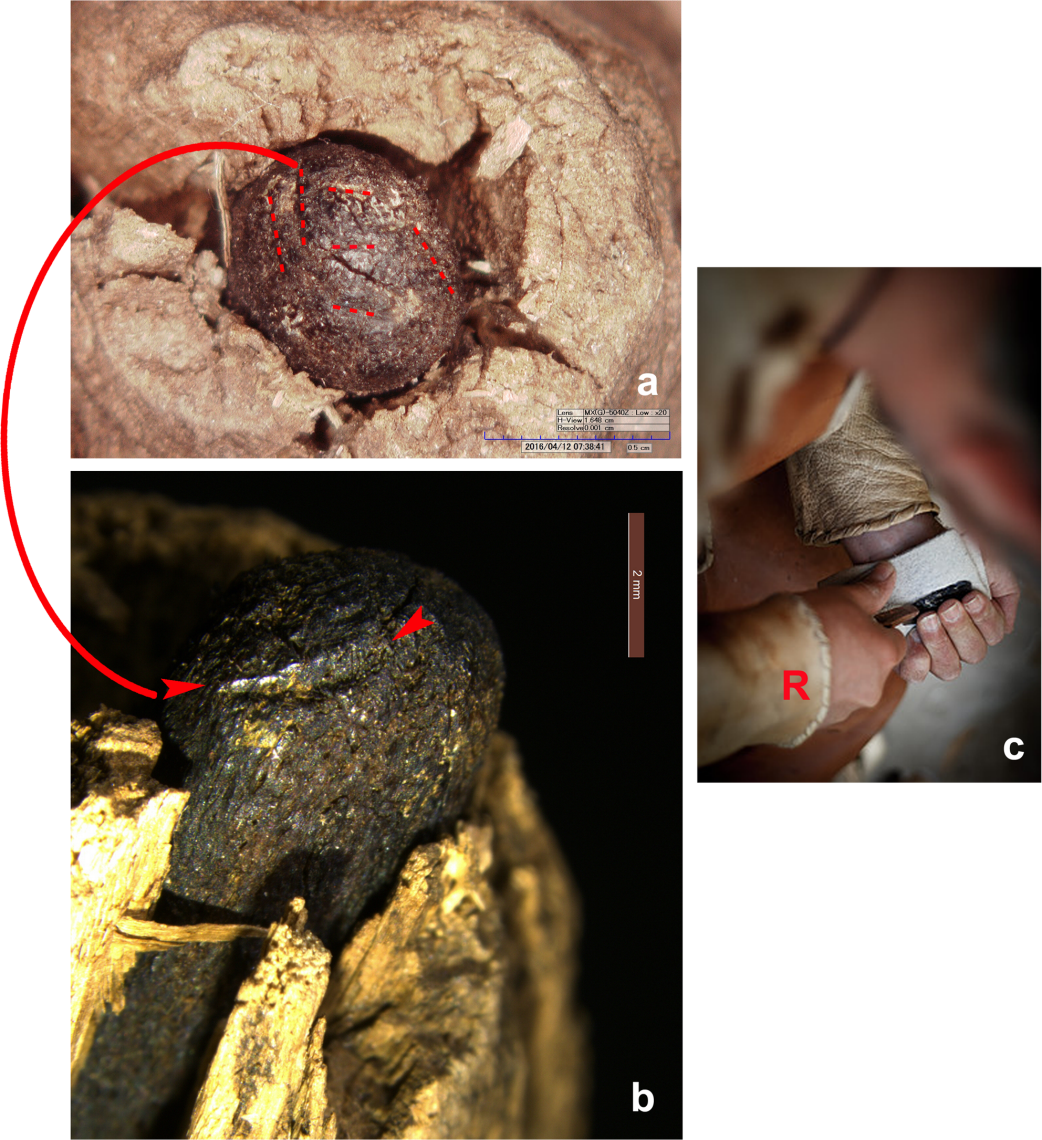
Most evident incisions from the use as pressure flaker visible on the retoucher. The “/”-orientation of the incisions were produced by a right-hander.

The tool appears as a fully functional pressure flaker. The blunt and rounded tip has the right shape, whilst a more pointed tip would crush when applying force by pressure. Length and thickness of the tool, 115 x 26 mm, are ergonomically appropriate. Therefore, the prospected repointing of the consumed tip through the trimming of the handle “like a pencil” [69] is not consistent with a long tool life: such operations would have shortened the shaft and worsened its ergonomic properties. A more correct maintenance would have been to tear the consumed spike out and to place a shim at the base of the canal. The wooden handle is intensely worn with several rounded and damaged parts. The apex of the antler tip bears small incisions caused by use. Most of them are distributed along the convex zone between the apex and the lateral surface: an expert knapper in fact, instead of working orthogonal to the flake edge, does orient his tool somewhat obliquely. All incisions on Ötzi’s retoucher are oblique with respect to the tool axes (Fig 18). Due to the fact that pressure flaking is lateralized work, the direction of the incisions depends on the handedness of its user. Experiments show that oblique incisions oriented according to the medial segment of a “Z”–as on Ötzi’s retoucher–are determined by a right-handed knapper. The black color of the spike extremity has been explained by exposure to fire or by having been soaked in birch-bark-tar [9, 62, 70]. No tar traces are present on the tool, so that the latter hypothesis can be rejected. The first one is not credible either, as a hardening of the antler over fire is not necessary and, on the contrary, diminishes its flaking properties. Antler becomes dry and starts to mineralize during the velvet loss of the living deer [71]. A further artificial drying would cause a loss of elasticity and bending strength causing the breakage of the collagen fibers. Seeing that the black coloring covers the incisions (Fig 18B), the blackening must have occurred after use, maybe through post-depositional chemical reactions.

Comparable deer antler spikes, though rare, are known in the Northern Italian Chalcolithic. Parallels have already been established with 4 artefacts from the eneolithic burial site Riparo Cavallino, Monte Covolo (BS) [72]. A similar spike was also found within an eneolithic burial site, Riparo Nogarole 2 in Trentino [73] associated with an arrowhead with a flat retouch.

## 4. Chert raw material

### 4.1 Arrowhead of arrow 12

**Fig 19:**
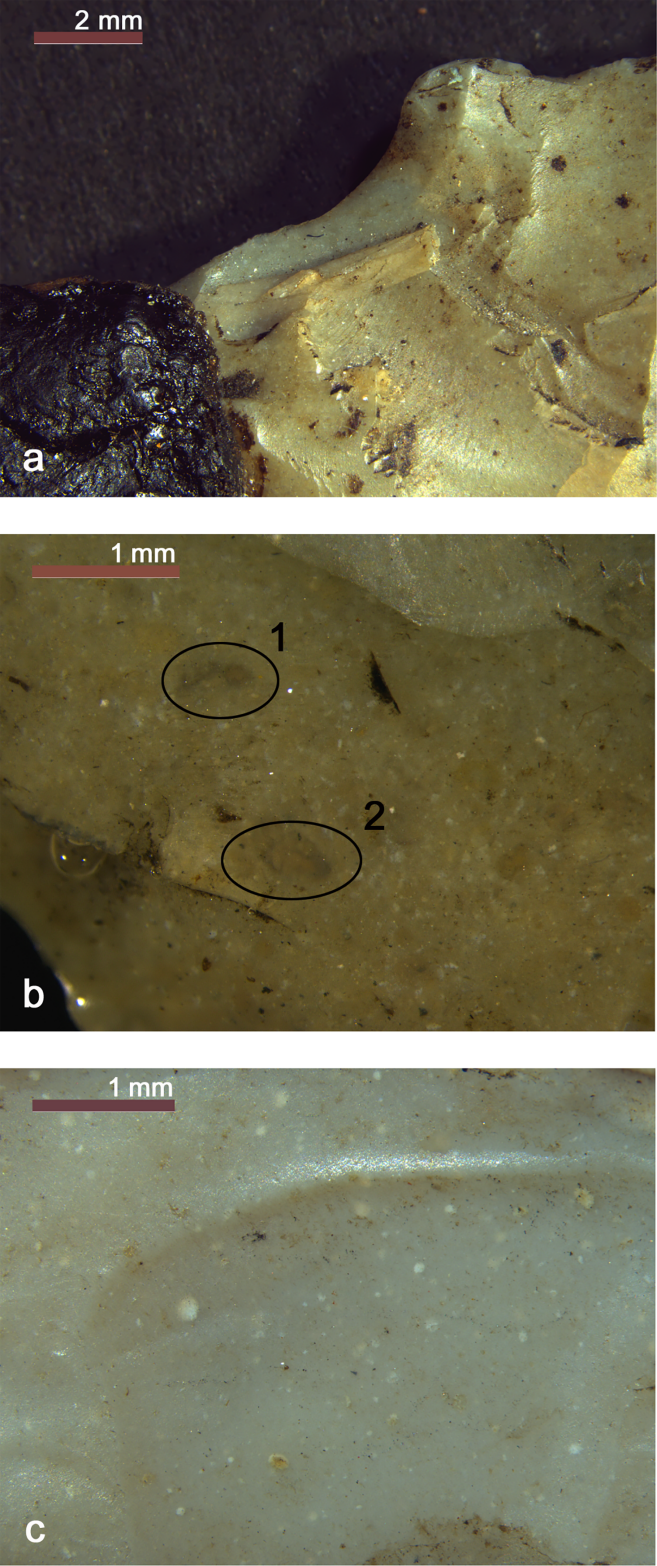
a-b) Arrowhead 12 (Scaglia Variegata Alpina, Trento Plateau). a) pale brown patina. The true grey to green chert color is visible in places; b) Microcrystalline chert matrix with planktic foraminifers - the species *Rotalipora cushmani* (1) and *Preglobotruncana* g*ibba* (2) are clearly recognizable - and radiolarians. Some microfossils have light reddish to brown fillings. c) Arrowhead 14 (Maiolica, Trento Plateau). Very clean and fine crystalline chert matrix incorporating whitish radiolarians.

Arrowhead 12 shows a yellow to brown patina which is more developed on face A, whereas the true color is grey to green (5Y 6/1 shading to 5Y 6/2) (Fig 4 and Fig 19A). The chert texture is very fine crystalline without evident limestone remains. There are no evident visible structures. It contains radiolarians and planktic foraminifers. The microfossils are either dark (grey microquartz fillings) or light (milky quartz but also calcite fillings). Among the planktic foraminifers there are clear Preglobotruncana and fewer Rotalipora specimens (Fig 19B). Some of the microfossil cavities have been filled by a reddish brown fine sediment (pelagic mud) and, locally, the siliceous matrix itself tends to reddish brown hues. These features indicate that the enclosing limestone is reddish-brown. It is classifiable as radiolarian and planktic foraminifers silicified wackestone [74]. The microfossil association clearly suggests middle Cretaceous (Cenomanian) age.

### 4.2 Arrowhead of arrow 14

Arrowhead 14 is made from a very homogeneous light grey chert with a slight brown patina (Fig 7). In close detail the cryptocrystalline light grey quartz matrix (5YR 6/2) incorporates tiny white elements (Fig 19C): 1) abundant and differently shaped radiolarians whose original opaline skeletons are preserved as mold fillings (microquartz, Fe-Mn oxides); 2) a few sponge spicules; 3) a few Proto-Globigerina foraminifers; 4) small limestone remains, not or only partially silicified. It is classifiable as radiolarian and Proto-Globigerina silicified wackestone [74]. This association dates the chert to lower Cretaceous (Valanginian—Hauterivian interval). The deposition took place in a deep and quiet environment where pelagic muds slowly sedimented on the ocean floor, far from continental detrital inputs.

### 4.3 Borer

**Fig 20:**
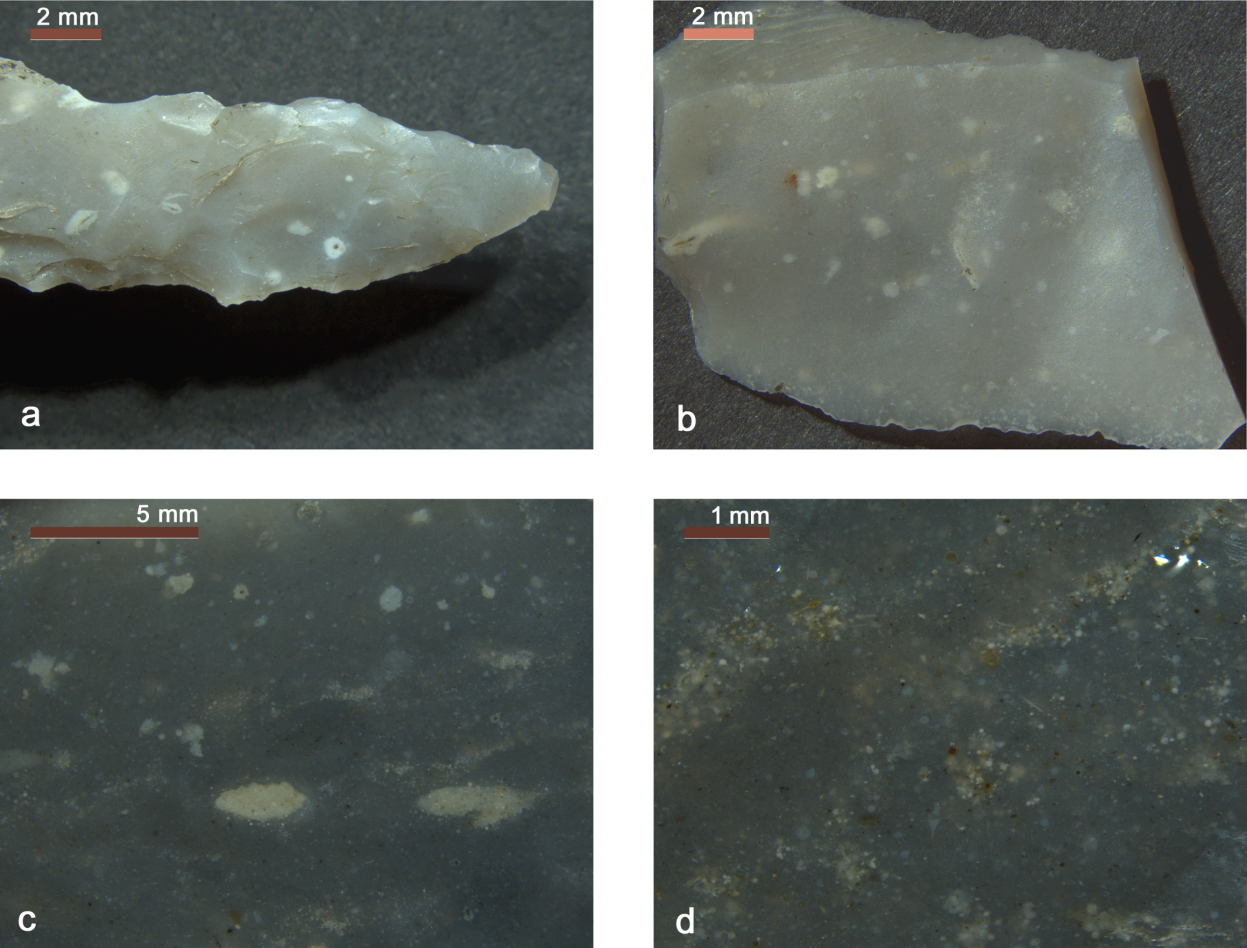
a-b) Borer and small flake (Maiolica, Trento Plateau). Fine crystalline chert matrix with radiolarians, a few sponge spicules and spotted limestone remains, sometimes surrounding green clay. Sparse iron oxides are common. c–d) Endscaper (Maiolica, Trento Plateau). Dark grey, fine crystalline chert matrix incorporating very well preserved radiolarians, sponge spicules and differently “digested” limestone remains. At places (d) these latter are almost completely silicified leaving only concentrations of calcite particles. There are also vague laminations.

Basically the chert type is the same as that of the arrowhead 14 (Fig 12 and Fig 20A) (color: 5YR 6/1 shading to 5YR 5/1). There are differences in both the amount and size of the limestone remains, but these features are quite variable, even in a single chert nodule. The brown to green hues sometimes visible in the center of the limestone remains are diagenetic clay mineral concentrations. This tool has also developed a light brown alteration patina. The rock is a lower Cretaceous radiolarian and Proto-Globigerina wackestone.

### 4.4 Small flake

The chert type of the small flake is very similar to the borer (Fig 14): both artefacts could even have been detached from the same block. Besides radiolarians, a few sponge spicules are also clearly visible and well preserved, in some cases highlighted by Fe-Mn oxide deposits (Fig 20B).

### 4.5 Endscraper

The chert type of the endscraper is darker and little more heterogeneous with respect to arrowhead 14, borer and small flake (Fig 9). Vague and often discontinuous laminations can be noted, underlined by limestone remains and/or microfossil alignments (Fig 20C and 20D). The microfacies is constituted by a cryptocrystalline dark grey quartz matrix (7.5YR 5/1 shading to 7.5YR 4/1) with radiolarians, sponge spicules and calcispheres. The microfossils have mainly a white look (milky microquartz fillings), secondarily they appear brown (calcite fillings) or red to black (sulfides fillings). The dark grey matrix hues and the presence of sulfides attest a deposition in a fairly anoxic environment. The chert features and the microfossil association suggest a lower to middle Cretaceous age (Barremian–Aptian interval).

### 4.6 Dagger

**Fig 21:**
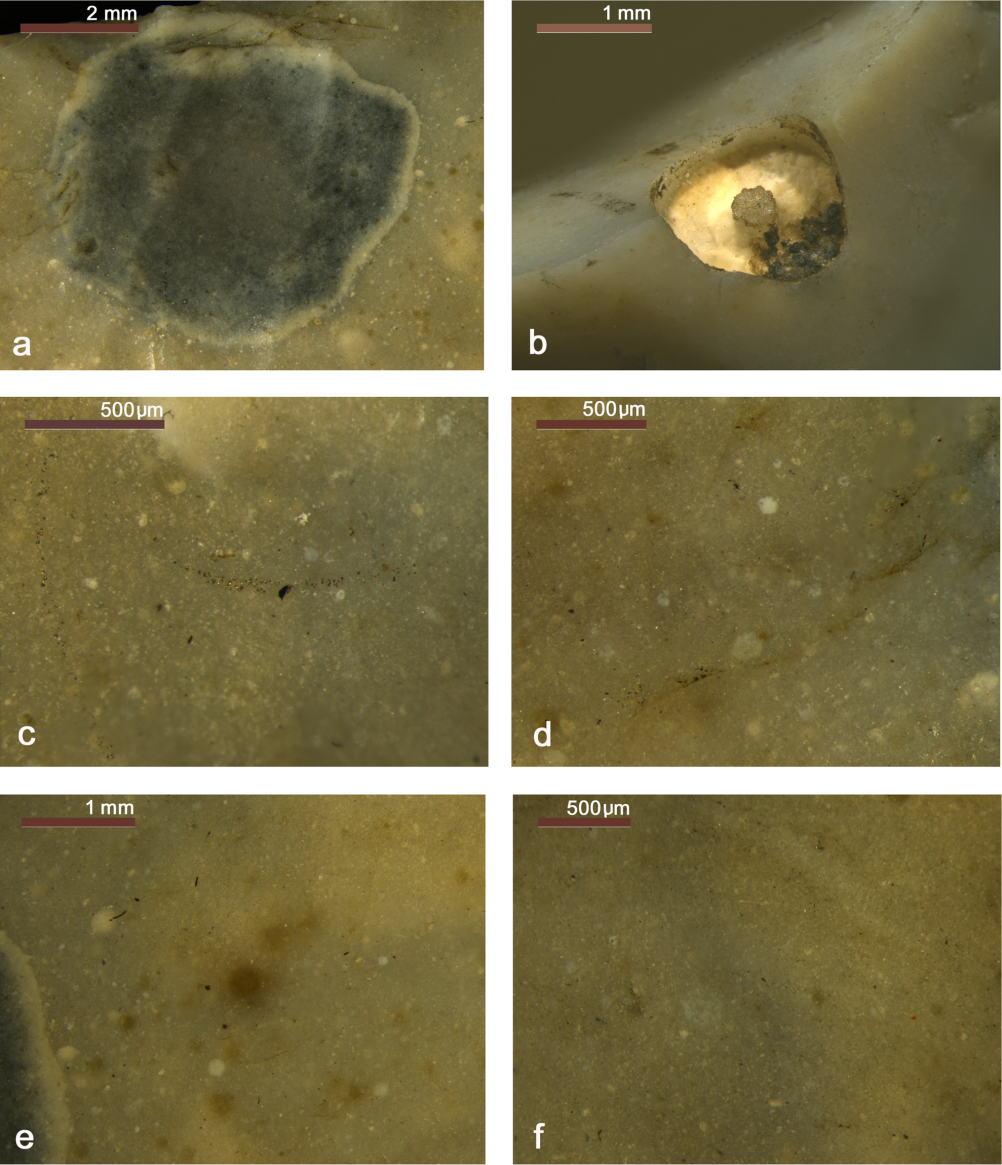
Dagger (Maiolica, Lombard basin). a) Bioturbation (trace fossil). The microfacies is similar to the surrounding sediment, but darker due to the organic matter enrichment; b) Benthic crinoid stem ossicle in correspondence with the apical fracture; c-d, f) Radiolarians, sponge spicules, pelagic crinoids (*Saccocoma*), calpionellids, holoturians sclerites in a microcrystalline quartz and calcite matrix. Fe-Mn oxide spots and organic matter remains also occur. The not fully silicified chert has a microdetrital look; **e)** Radiolarians, partly filled with ferruginous mud, in a microcrystalline quartz matrix spotted by several tiny brownish calcite particles.

The chert of the dagger is the most particular due to the richness of evidences (Fig 2). Two of the chert features are clearly visible also with the naked eye (Fig 21A and 21B). The first is a dark circular, 5 mm large stain, surrounded by a white border. It could be either an intraclast, but more probably a trace fossil (burrow filled with sediment enriched with organic matter). The other feature is a crinoid columnal element visible on the apical fracture. It belongs to the stem of a sea lily (Echinodermata) which live attached to the sea bottom in their adult form. Most benthic crinoids live in shallow waters, but often they are transported to the bottoms by turbidites. Going into more detail, the chert cryptocrystalline matrix looks grey (5YR 6/1 shading to 5YR 6/2), spotted by several tiny calcite crystal particles: this denotes a not full silicification of the former limestone. A few white to light grey limestone remains and intraclasts (fine crystalline micrite mud) are locally quite evident and often aligned (Fig 21C). The microfossils are mainly represented by radiolarians, calcispheres, calpionellids and a few sponge spicules (Fig 21C and 21D). Some of the radiolarians have been clearly filled by reddish brown clay (terrigenous inputs) (Fig 21E). There are also several fragments of pelagic crinoids (Saccocoma) (Fig 21C, 21D and 21F). The chert has a micro-detrital texture deriving from the silicification of a resedimented layer in the sea bottoms (turbiditic horizon). This explains the presence of benthic crinoids, intraclasts and trace fossils. The rock is classifiable as silicified bioclastic packstone [74]. The presence of calpionellids and Saccocoma indicates an upper Jurassic age (Tithonian).

### 4.7 Critical comment on previous raw material studies

With respect to former studies of the Iceman’s lithic assemblage by A. Binsteiner [75–78] some analogies but also significant differences, especially in the description of the microfacies, could be found. The lithotypes of the Iceman’s assemblage belong to the so-called replacement cherts deriving from a diagenetic substitution of former limestones with silica of mainly biogenic origin (radiolarians and sponge spicules). This substitution process, taking place on the ocean bottoms, is almost always incomplete, thus the Iceman’s cherts have a “brecciated” look where the clasts represent unsilicified portions of the original limestones. Many of these diagenetic limestone remains have been wrongly described by Binsteiner [78] as bioclasts or macrofossils. In contrast, the present study found only few macrofossils for the dagger, whereas the chert microfacies of the assemblage are nearly entirely constituted by planktonic associations (radiolarians, foraminifers, calpionellids, calcispheres, pelagic crinoids). The same outcrops, which Binsteiner uncritically proposed as the probable source of the Iceman’s tools (Ceredo, Verona), differ significantly from his descriptions.

### 4.8 Discussion of the chert raw material

**Fig 22:**
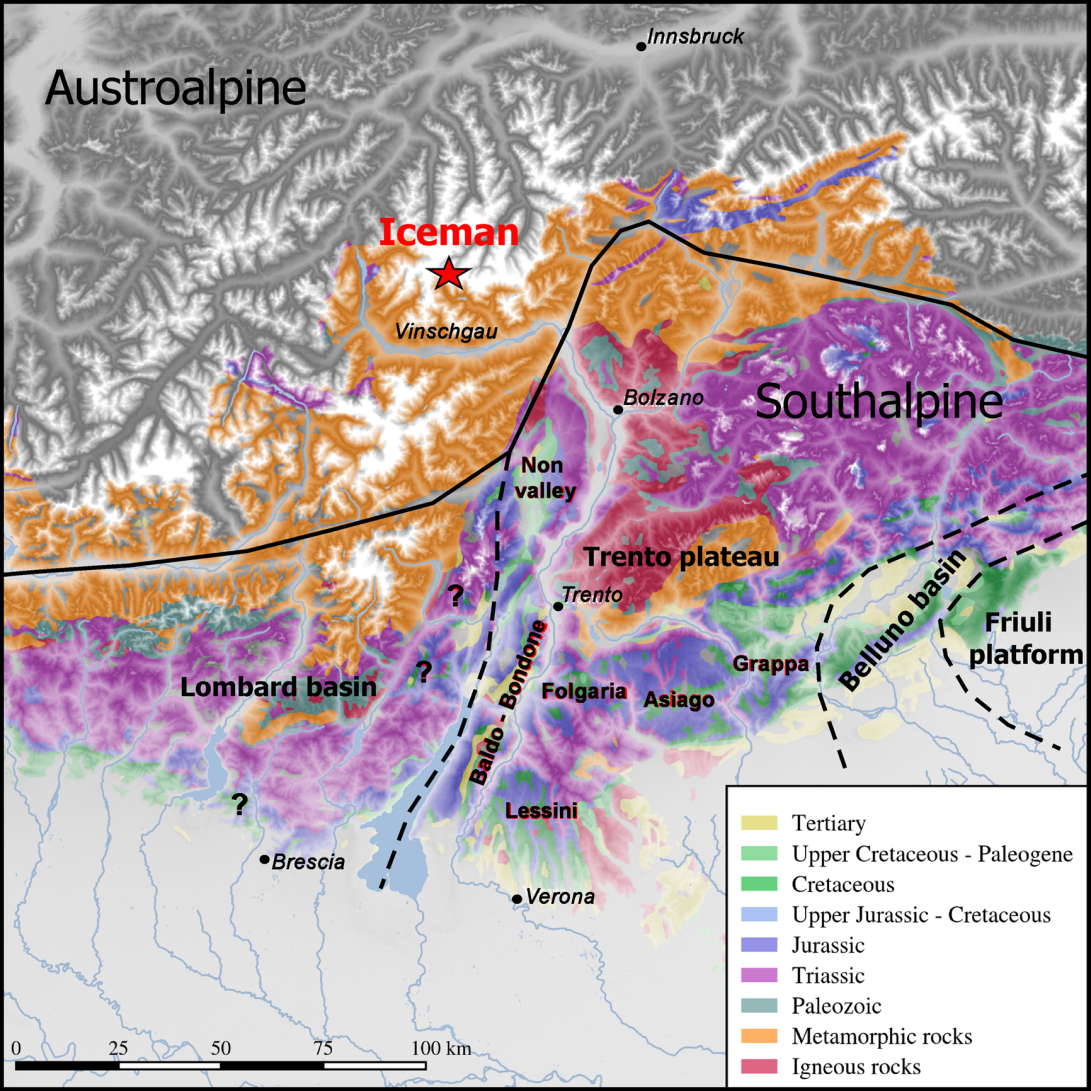
Geological outline of the Austroalpine and Southalpine series, separated by the Periadriatic line. The Iceman was found in an area where the substratum is fully metamorphic (Austroalpine). The lithic tools come from the Southalpine: 5 from the Trento Plateau series, most probably from the outcrops situated near the Adige valley (Non Valley, Baldo-Bondone, Lessini), whereas the provenance of the dagger is probably from the eastern slope of the Lombard Basin.

From a paleogeographic viewpoint the recovery site of the Iceman lies in the area between the Austroalpine and Southalpine domains (Fig 22). The features of the Iceman’s cherts though leave no doubt about the fact that their provenance must be sought in the Southalpine series. Their characters correspond in fact with the very fine and almost pure pelagic silicified calcareous muds which are very common in the Maiolica-Scaglia Variegata Alpina (upper Jurassic-middle Cretaceous) of the area. These formations, although deposited over a wide territory, show distinctive regional features, mainly determined by the articulated substratum where they have been deposited: submarine plateaus (see Trento plateau) and bottom basins (see Belluno and Lombard basins) [79–80]. Such articulated palaeogeography affected the sedimentary patterns in terms of thickness of pelagic muds, oxygenation, diagenesis, amount of terrigenous inputs and of resediments [81–82] so that, on a wide scale, even the cherts can be distinguished [44].

**Fig 23:**
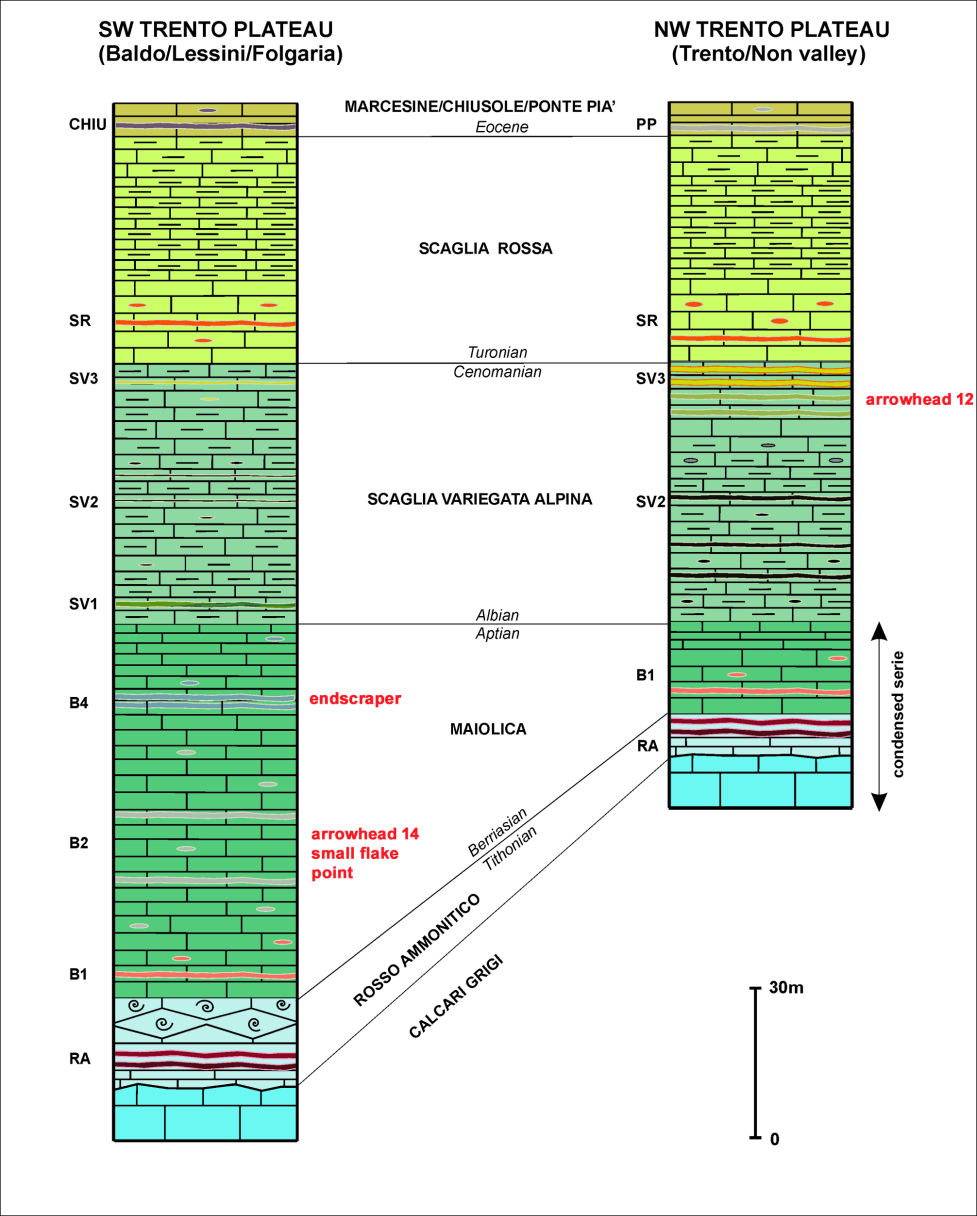
Schematic drawing of the upper Jurassic-Eocene sedimentary series of the western Trento plateau. The NW sector of the area (right) characterizes for upper Jurassic gaps or very condensed series. The Iceman’s tools are indicated in correspondence with the comparable chert types.

On the base of the observed petrographic features, five of the Iceman’s tools - endscraper, borer, flake and both arrowheads - are very likely made from cherts collected from the Trento plateau outcrops. The Trento plateau, a submerged raised block, had an almost pure pelagic sedimentation during the late Mesozoic (Upper Jurassic–Upper Cretaceous). The sedimentary sequence characterizes for frequent cherts in its entire interval, with distinctive features according to variable sedimentation patterns (on a wide scale) in different stratigraphic layers. Locally the depositional features may vary due to the presence of palaeogeographic heights, in correspondence of which condensed series were deposited, as observed in the Non Valley **(**Fig 23) [42–43, 45–46].

At a close analysis with the Trento plateau series, arrowhead 14, borer and small flake belong to the Maiolica and correspond to the B2 chert type distributed in the middle part of the formation. The endscraper belongs to the Maiolica as well, but to the B4 chert type distributed in the upper part of the Maiolica. Regarding the possible area of provenance, the cited chert types are frequent in the Monti Lessini, but comparable cherts are also known from several other localities such as the Baldo-Bondone chain, Folgaria, Asiago and Grappa Mountains. Traces of cortex or natural surfaces, which could give further information about the chert gathering contexts (from outcrops, soils or detritus) are absent from all artefacts.

Things are different for arrowhead 12. Compatible cherts, available in exploitable quantity and proper knapping quality, are usually not frequent and found in spots. An exception is represented by the Non Valley NW of Trento where the Cenomanian Scaglia Variegata Alpina formation contains very abundant, fine crystalline and homogeneous cherts **(**Fig 23**).** The Iceman’s arrowhead 12 is very similar in its fine details to the SV3 chert type of these outcrops, and in particular with the green variant [43].

The features of the dagger suggest a different depositional environment from the other artefacts. The chert surely belongs to the Maiolica formation, but the presence of resediments from shallower areas denotes a depositional environment on a paleoslope. Even if it is difficult to exactly state the palaeogeographic location of the exploited outcrops, the features fit with the area placed between the edge of the western Trento plateau and the eastern Lombard basin where these sedimentation patterns are very common. From a geographic point of view the area lies between the Garda and the Iseo lakes within which comparable chert outcrops are situated in the Southwestern Trentino and on the Brescia foothills (Fig 23, question marks).

## 5. Typological aspects and comparisons

### 5.1 Dagger

The appearance of bifacial flint daggers in Northern Italy is dated to the first half/middle of the 4^th^ millennium BC [83–84], roughly contemporary with the first bifacial daggers in Central Italy [85–87]. At the same time bifacial daggers are attested in Central-Eastern Switzerland and Southern Germany, comprising examples imported from Northern Italian production centers [88–90].

**Fig 24:**
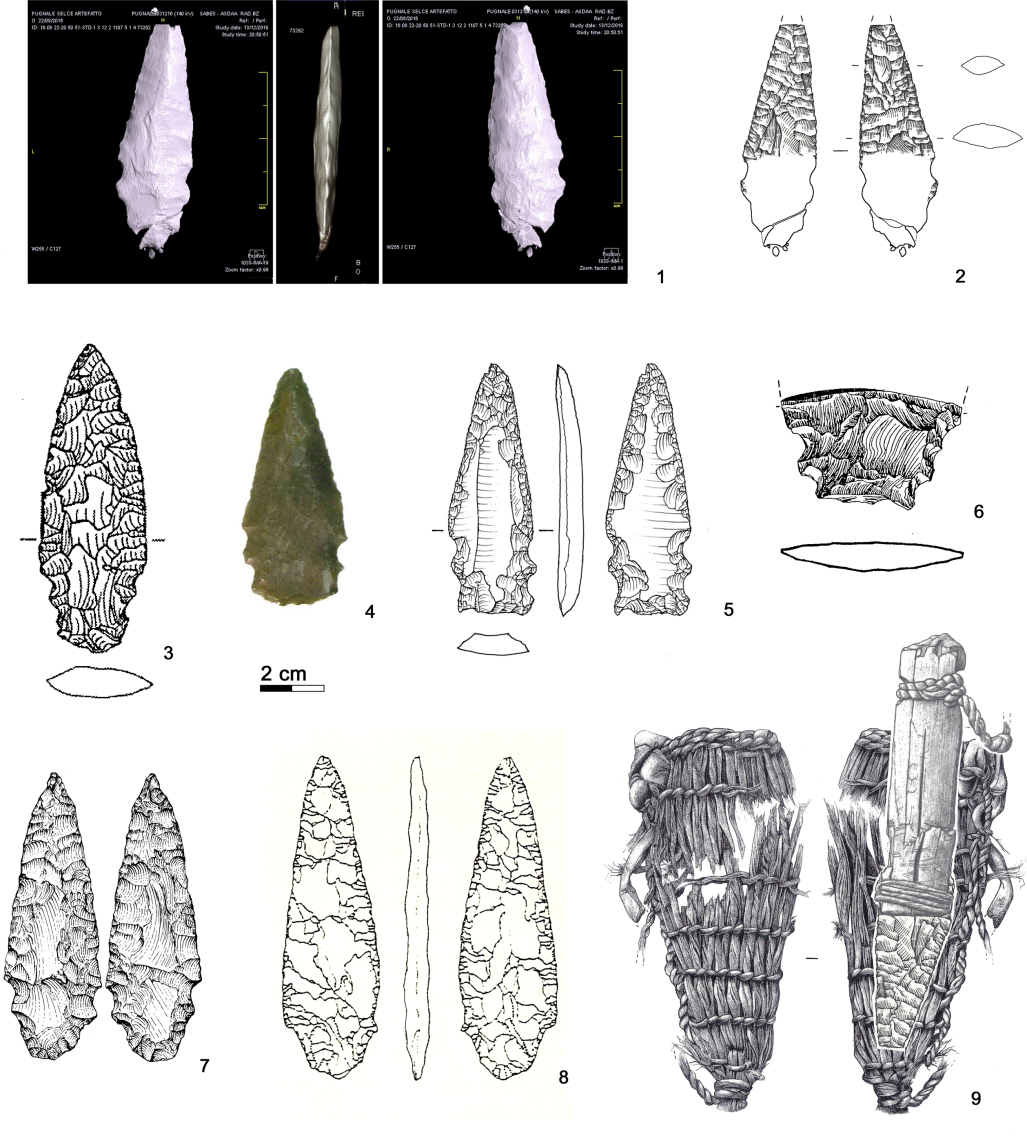
Dagger. **Typological comparisons.** 1–2) CT-image and drawing of the Iceman dagger; 3) Wallhausen; 4) Ludwigshafen-Seehalde; 5) Zürich-Otelfingen (Kantonsarchäologie Zürich); 6) Sipplingen-Osthafen; 7) Buca delle Fate, Cardoso; 8) Brentonico; 9) Iceman dagger in bast scabbard.

The bifacial dagger of the Iceman is characterized by an asymmetric triangular blade with anciently broken tip. The rectilinear blade edges end with lateral notches which hold the fixing of the handle (Fig 24, 1–2). Below the notches there is a distinct, slightly convergent tang. Its base was fractured during discovery. The CT images show that the base fragments have not been greatly displaced; therefore the base could have been trapezoidal. The Iceman’s dagger sized 69 x 24 x 7.7 mm (even with intact tip not longer than 90 mm) is small with respect to most eneolithic daggers. The edge resharpening (see chapter 3.1) and the size of the bast scabbard measuring 120 x 45 mm suggest that the initial size of the tool was somewhat bigger (Fig 24, 9).

The most distinctive and enigmatic feature of the Iceman’s dagger blade is its proximal part analyzable only by means of the CT images. With respect to the other notched daggers the tool has been compared with in the past (Remedello Sotto tomb 97 [83]; Grotta da Prima Ciappa Superiore [91]; Arbon Bleiche 3 [92]; Piran [93]), its shape is more complex. Egg and Spindler [9] interpreted the presence of two hafting devices, tang and notches, as the result of a first hafting followed later by a second one. According to such a hypothesis, the original shape before reworking could have been a dagger with distinct tang, similar to the daggers found for example at Brentonico (Trento) (Fig 24, 8) [94]. Recycling and rehafting of daggers was indeed a common habit, especially in areas far from high-quality chert outcrops [89, 95–97].

Advancing another hypothesis, the dagger base could have been conceived with its actual shape. Examples with notches and tang or with double notches are attested. Three daggers with double notches, made apparently from Northern Italian chert, are found at lake dwellings of Lake Constance (Southern Germany). The tools from Ludwigshafen-Seehalde (Fig 24, 4) and from Wallhausen, a 37^th^-29^th^ century settlement (Fig 24, 3), were found out of context, whereas the base fragment of Sipplingen-Osthafen (Fig 24, 6), recovered in old excavations, is dated to the beginning of the 3^rd^ millennium BC [89, 98–99]. Analogous forms are found also among blade daggers of western tradition, as shown by the surface find with double notches from Otelfingen near Zürich (Fig 24, 5) [97, 100]. Double notches are displayed also by a strongly reworked radiolarite dagger from Zürich Kanalisationssanierung, layer 4, dendro-dated to the 33^rd^ century BC [97]. The most evident similarity with the Iceman’s tool can be made with a Central Italian dagger from Buca delle Fate at Cardoso in Tuscany, an eneolithic burial cave (Fig 24, 7) [101]. No striking comparisons with notched daggers from North-eastern Italy can be proposed to date, although the region, well known for its eneolithic chert extraction and production centers [102–103], yielded numerous bifacial daggers attributed to the Copper Age [84, 95, 104]. In general, the corpus of the daggers with lateral notches displays a high typological and morphological variability, which hardly allows any precise comparisons with the Iceman’s tool.

### 5.2 Arrowhead of arrow 12

**Fig 25:**
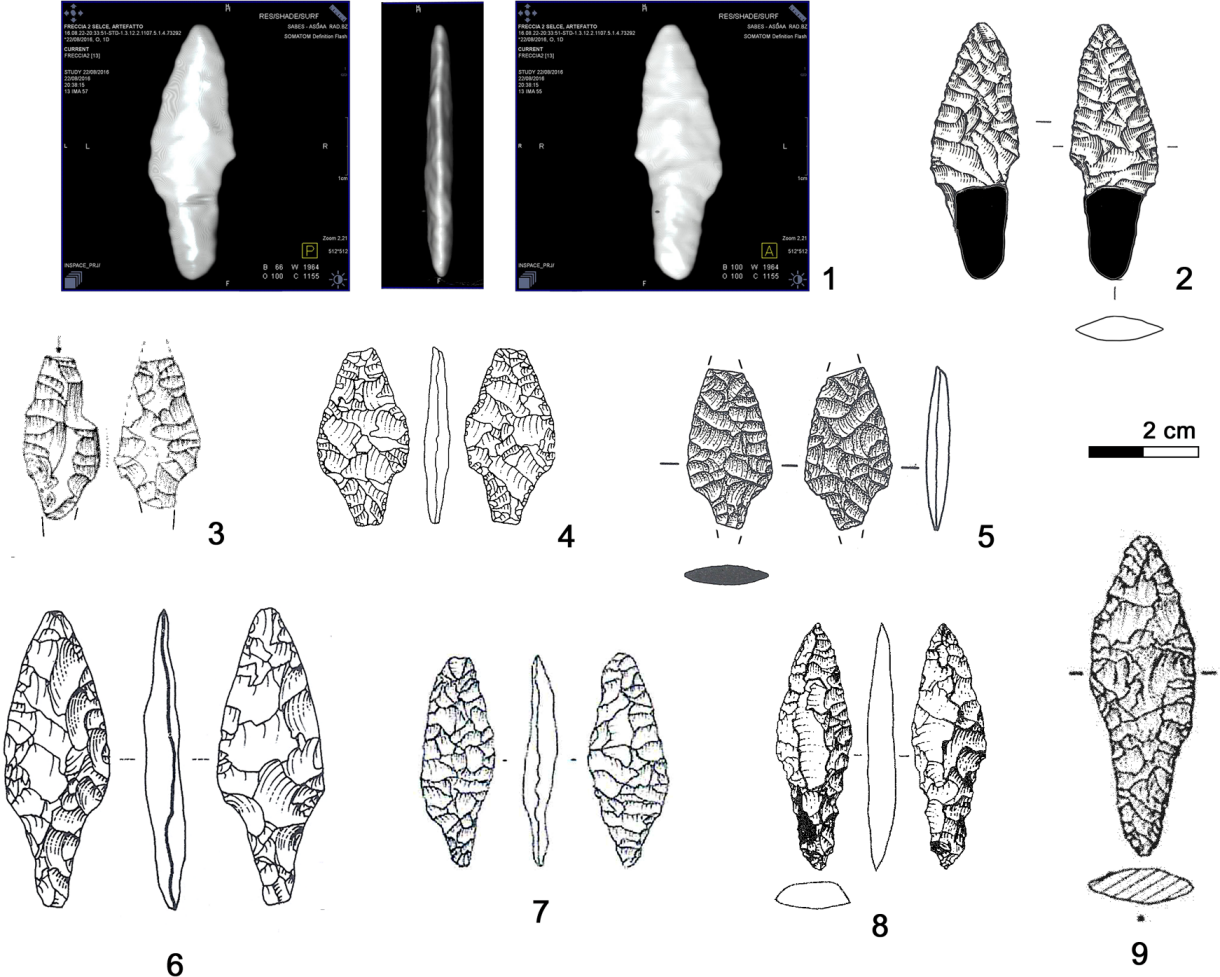
Arrowhead 12. **Typological comparisons.** 1–2) CT-image and drawing of the Iceman’s arrowhead; 3) Riparo Valtenesi; 4) Isera La Torretta; 5) Schlern Roterdhang; 6) La Nasa di Cerro; 7) Povegliano Veronese; 8) Steinhausen-Sennweid; 9) Petit-Chasseur III.

Arrow 12 is armed with a tanged arrowhead made with flat, covering, bilateral and bifacial retouch (Fig 25, 1–2). The piece, which measures 46 x 16.3 x 4.7 mm, has a broken tip, a nearly continuous lateral profile (the concavity on the right edge is accidental) and a large convergent tang with rounded base. The tang is actually shorter than how it has appeared in previously published drawings [9, 35, 105]. The piece is classifiable as simple foliate tanged point [106].

Foliate tanged arrowheads are typical of the Italian Copper Age, but despite the extended *corpus* available for Northern Italy, arrowheads with comparable shapes are rare. Considering the dated contexts, they are found at the settlement Isera La Torretta, US 32 = 35 (Trento) (Fig 25, 4) [107] at 4801±37 BP (DSH4098 3654–3519 cal BC) and at the burial site Riparo Valtenesi, Northern Platform (Brescia) (Fig 25, 3) [108] at 4105±65 BP (2879–2491 cal BC). Similarities are found with a find from the settlement Povegliano Veronese (Verona) (Fig 25, 7) [109] containing recent Neolitic and Copper Age material. Surface findings are known from the mountain site Schlern Roterdhang (Bozen) (Fig 25, 5) [110] and from La Nasa di Cerro (Verona) (Fig 25, 6) [111]. The sporadic comparisons found in Switzerland refer to dolmen MXII 5B of Petit-Chasseur III (Valais) (Fig 25, 9) [112] in use from 4390±80 BP to 4055±65 BP (3339–2891 to 2871–2466 cal BC), and to the lake dwelling Steinhausen-Sennweid (Zug) (Fig 25, 8) [113–114], dendro-dated to 2869–2859 and 2764–2762 BC.

Southern Alpine comparisons, found in burial-ritual contexts and settlements, occur in a rather limited area of South Tyrol and neighboring provinces at least between the 37-36^th^ and the 29-26^th^ century cal BC. In Swiss sites, both burials and settlements, they are attested between 34-30^th^ century cal BC to the 29-25^th^ century cal BC.

### 5.3 Arrowhead of arrow 14

**Fig 26:**
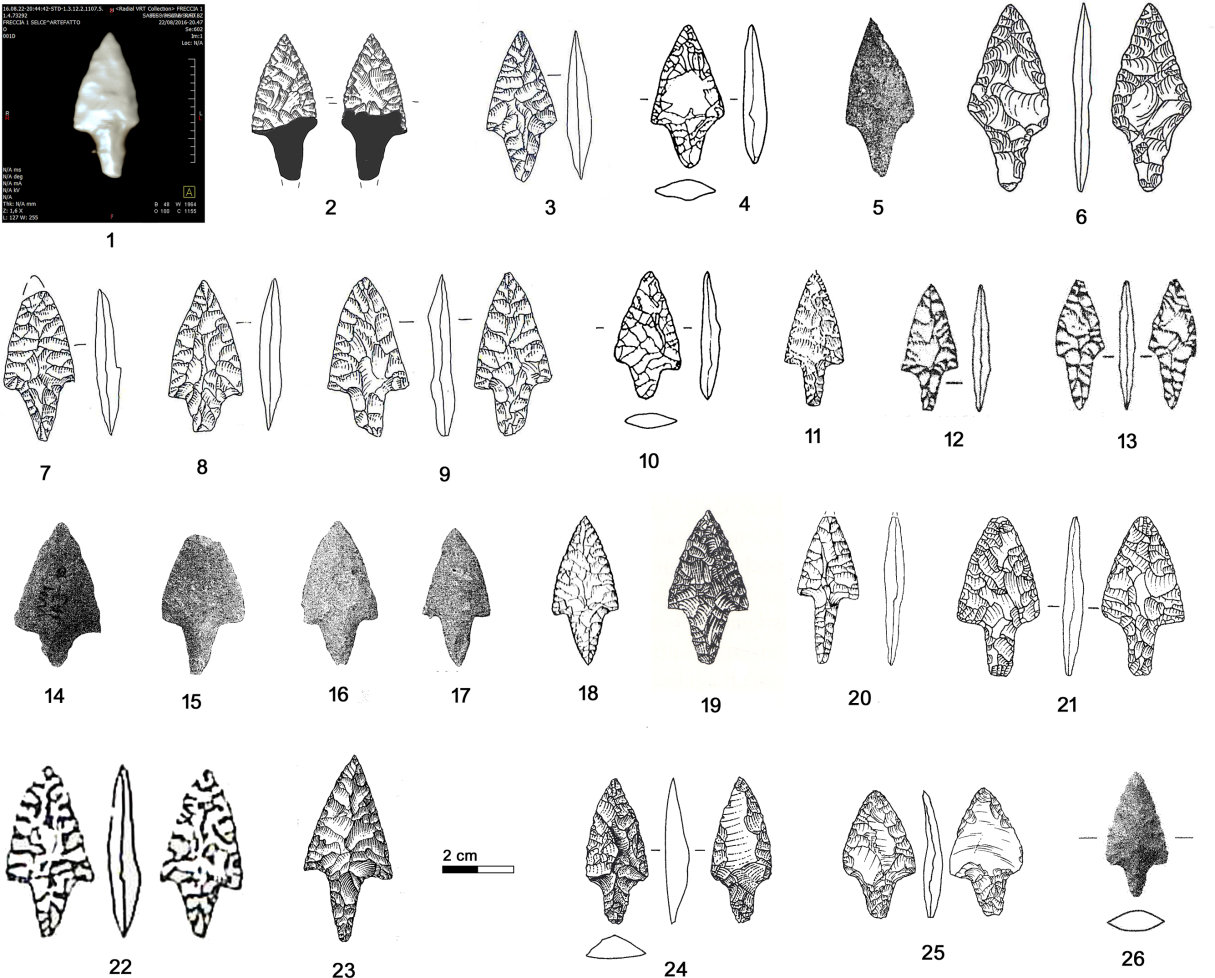
Arrowhead 14. **Typological comparisons.** 1–2) CT-image and drawing of the Iceman arrowhead; 3, 7–9) Spilamberto; 4–5, 10) Remedello Sotto; 6) Scalucce di Molina; 11) Monte Loffa; 12) Palù di Cordignano; 13) Montello; 14–17) Vahrn; 18) Casarole; 19) Monte Aiona/Prato Mollo; 20) Sanguani; 21) Isera La Torretta; 22) Buca d’Andrea; 23) Ca’dei Grii; 24) Steinhausen-Sennweid; 25) Zürich-Kanalisationssanierung (Kantonsarchäologie Zürich); 26) Uebeschi-Subelmoos.

Arrowhead 14 is made with flat, nearly covering, bilateral and bifacial retouch and measures 38 x 18.5 x 4.5 mm (Fig 26, 1–2). The asymmetric triangular point has convex borders and straight shoulders. The converging tang is broken and the fragment has not been found. The piece is classifiable as foliate point with tang and shoulders [106].

Numerous analogies are found in Northern Italian contexts: as grave goods at the well dated ritual and burial site Vahrn (Bozen) (Fig 26, 14–17) [31] in use around 4764±33 BP and 4728±33 BP (3640–3383 and 3635–3377 cal BC), at Remedello Sotto (Brescia), graves 40 and 41 (Fig 26, 4–5, 10) [35, 115] and at Spilamberto (Modena), I°, grave a1 and X°, graves 9 left, 16 and 17 (Fig 26, 3, 7–9) [116] as well as in burials of the Lessini Mountains (Verona), especially Scalucce di Molina, Monte Loffa and Casarole [104, 117] and in the Verona plain, at Sanguani [104] (Fig 26, 6,11,18 and 20). Similar arrowheads are known from the burial caves of Buca d’Andrea (Bergamo) and Ca’ dei Grii (Brescia) (Fig 26, 22–23) [118–119]. A similar arrowhead is found at Isera La Torretta settlement, US 9 (Fig 26, 21) dated *post* 4607±39 BP (3518–3126 cal BC) [107]. Comparisons from surface findings exist at Monte Aiona/Prato Mollo (Genova) (Fig 26, 19) [89], where nearby sondages gave the datings 4300±60 and 4130±60 BP (3091–2698 and 2884–2501 cal BC), as well as at Palù di Cordignano and at Montello (Treviso) (Fig 26, 12–13) [120].

Arrowheads with similar shape, though not with covering retouch, are found in the Swiss final Neolithic settlements Steinhausen-Sennweid (Zug) (Fig 26, 24) [113–114], dendro-dated between 2869–2859 and 2764–2762 BC, and Zürich Kanalisationssanierung, layers E-A (Fig 26, 25) [121–122] dated between 2749–2700 and 2700–2675 BC. The arrowhead from Uebeschi Subelmoos (Bern) (Fig 26, 26) [123] is without context.

Arrowhead 14 represents a very common variety in Northern Italy, where it is often associated with burials, the prevailing Copper Age contexts of the region. It was in use at least from the 37-34^th^ to the 29-26^th^ century cal BC. The same variant is found in Central Italian cemeteries [124] showing its wide spread. The comparable forms found in Central Switzerland date to the 29-27^th^ century BC.

### 5.4 Arrowhead embedded in the Iceman’s shoulder

**Fig 27:**
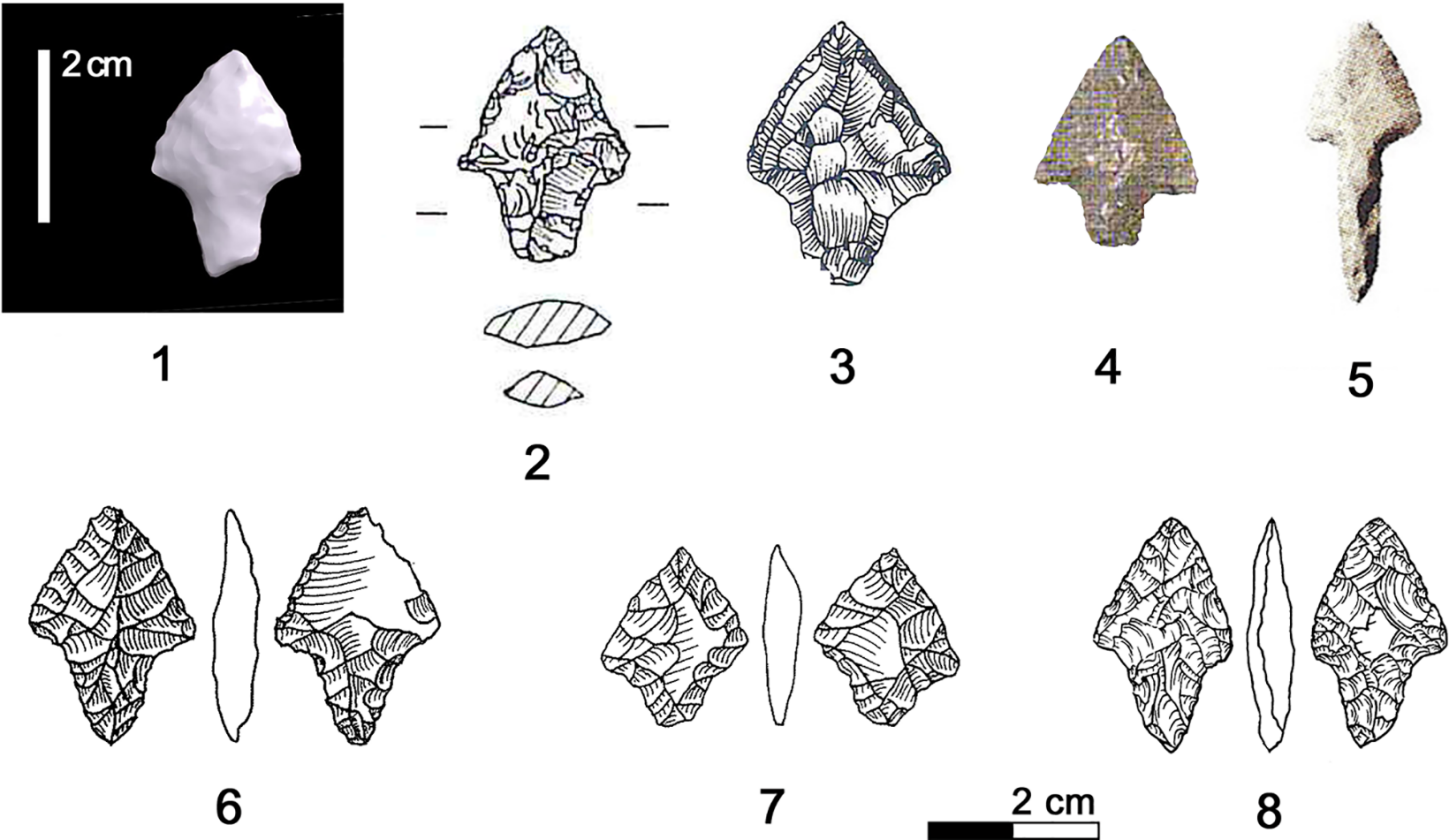
Arrowhead stuck in the Iceman’s shoulder. Typological comparisons. 1) CT-image of the arrowhead; 2) Volongo; 3) Altino Vallesina; 4–5) Vahrn; 6–7) Cham-St. Andreas; 8) Zürich- Kanalisationssanierung (Kantonsarchäologie Zürich).

Judging from the CT image, the arrowhead still in the Iceman’s shoulder is also made with flat bifacial retouch (Fig 27, 1). Sized 26.2 x 17.9 x 4.8 mm it is exceptionally small. The point with slightly convex borders and short convergent shoulders forms an isosceles triangle. The oblique termination of the large convergent tang could be a facture, but not from impact. Also this piece appears as a foliate point with tang and shoulders [106].

In Northern Italy small arrowheads of more or less comparable morphology are found at Vahrn (Bozen) (Fig 27, 4–5) [31] (datings above). They are known as grave goods at Volongo (Cremona), grave 1 (Fig 27, 2) [125] attributed to the first half of the 3^rd^ millennium BC. Surface finds come from Altino Vallesina near Venice (Fig 27, 3) [126]. Comparable small arrowheads from Central Switzerland are found at Zürich Kanalisationssanierung, layers E-A (Fig 27, 8) [121] dated to 2749–2700 and 2700–2675 BC and at Cham-St. Andreas (Zug), in the humus (Fig 27, 6–7) [127]. This latter lake dwelling inhabited from the 41^st^ century BC onwards, gave dendro-datings from 2704–2681 BC and around 2550 BC, as well as uncertain ones from 3141–3133 BC. The smaller item is apparently made from Lessinian chert.

In Northern Italy comparable arrowheads of small size were sporadically produced starting from the 37-34^th^ century cal BC and continue during the 3^rd^ millennium. In contrast, the most similar items from Central Switzerland date between the 28-26^th^ century cal BC.

### 5.5 Considerations about the arrowheads

In Northern as well as in Central Italy the tradition of tanged arrowheads with flat retouch, firstly with plano-convex and later with biconvex cross-section, exists from around 4800–4700 cal BC and lasts throughout the Copper Age [128]. Their production becomes intensive from the mid-4^th^ millennium onwards, as attested not only by the findings but also by quarries and arrowhead production centers in Liguria and Tuscany [129–130]. The three examined arrowheads are consistent with forms in use by the different cultural groups living in Northern Italy between the end of the first half of the 4^th^ to the second half of the 3^rd^ millennium (see also [9, 35, 105, 131]). North of the main Alpine divide, in Switzerland, rare tanged arrowheads appear after 3500 BC, and become frequent only from the 30-29^th^ century onwards [114, 132]. This makes it highly probable that the archer who shot the Iceman was of Southern Alpine provenance [35, 131]. Although possessed by a single person, the Iceman’s arrowheads show strong differences: arrowhead 12 which might be of a more local tradition, in contrast to the very common shape of arrowhead 14, which could even originate from standardized productions and organized trade.

### 5.6 Endscraper

**Fig 28:**
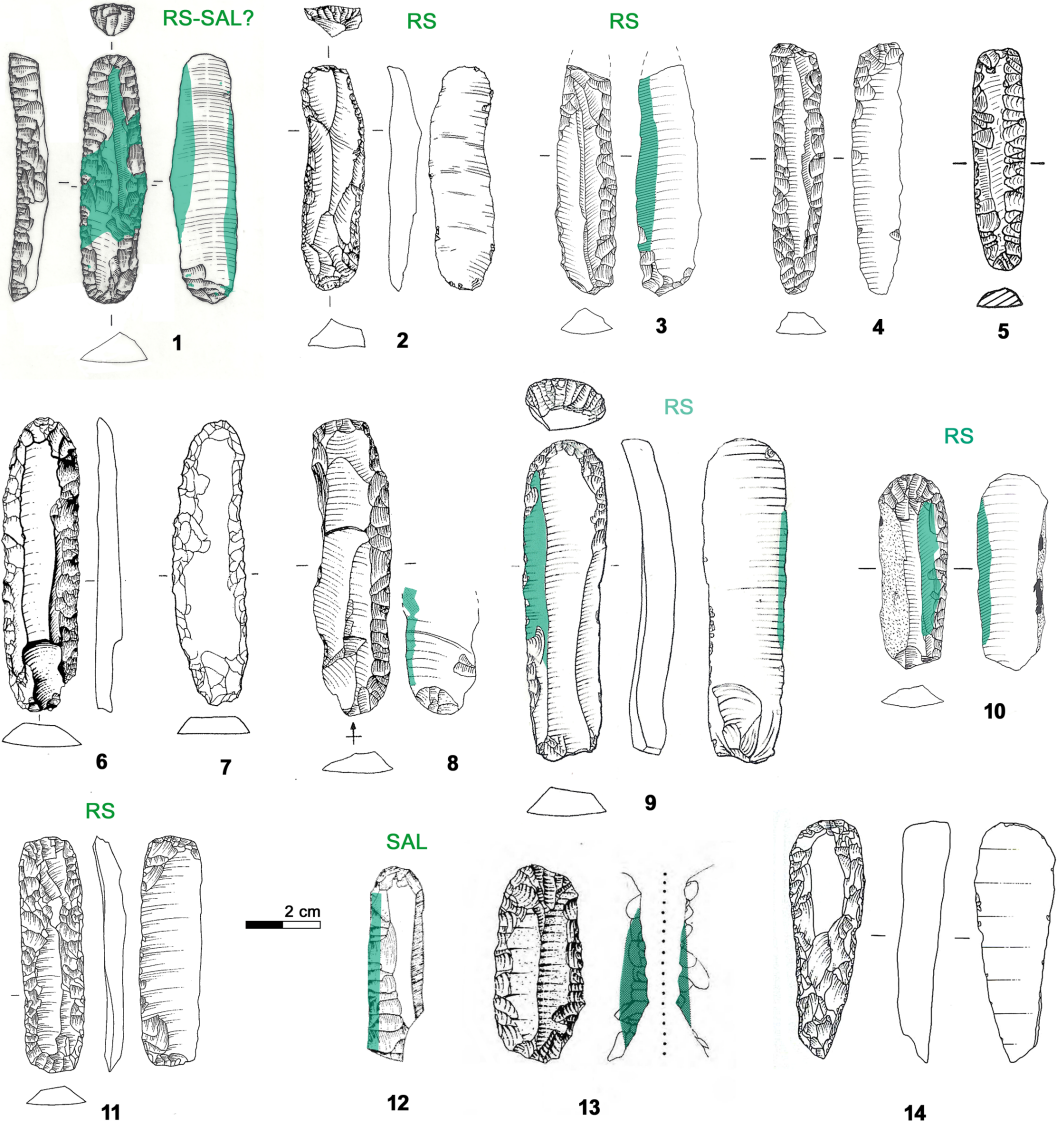
Endscraper. **Typological, morphological and use-wear comparisons.** 1. Iceman’s tool from Tisenjoch; 2. Zug-Vorstadt; 3–4,10 Arbon Bleiche 3 (Amt für Archäologie Thurgau); 5. Feldmeilen-Vorderfeld (Kantonsarchäologie Zürich); 6. Steinhausen-Sennweid; 7. Twann-Bahnhof; 8. Ermatingen-Westerfeld (Amt für Archäologie Thurgau); 9. Zürich-Parkhaus Opéra; 11. Pfäffikon-Burg (both Kantonsarchäologie Zürich); 12. Riparo Valtenesi; 13. Riparo Balm Chanto; 14. Arco-Linfano. Green areas = gloss; RS = resharpened; SAL = strike-a-light.

The tool indicated as “long chert blade” by Egg and Spindler [9] is classifiable as a carinated long endscraper with bilateral simple to superelevated retouch [133]. The proximal end of the 66.6 x 18.2 x 8.3 mm sized instrument is modified by simple direct and by flat inverse detachments (Fig 28, 1). Going beyond a merely typological classification, the repeatedly resharpened blade tool served mainly as knife to cut siliceous plants (cf. chapter 3.4).

Similar tools are not frequently attested in the Northern Italian Copper Age. Carinated endscrapers are known amongst old findings from Arco-Linfano (Trento) [134], a cache attributed to the Final Neolithic to the Copper Age (Fig 28, 14). Endscrapers with luster from the rockshelter Balm ‘Chanto (Torino) (Fig 28, 13) [135] dated to 4090±70 BP and 4010±60 BP (2873–2488 and 2855–2346 cal BC) can be cited as items following the same tool concept. Morphological and dimensional comparisons can be made with instruments classified as knives, as shown by the blade with lateral retouch and luster from the burial site Riparo Valtenesi (Brescia) (Fig 28, 12, reused as a strike-a-light) [108] dated to the first quarter of the 3^rd^ millennium [136]. Heavily used blade tools with long functional life, though rare, are a typical component of the Central Italian Final Neolithic and Copper Age [137–138]. They are interpreted as sign of a high mobility and as personal belongings.

Interestingly the Iceman’s endscraper shows strong parallels, also in size and morphology, with blade tools from Swiss and South German lake dwellings of the Horgen culture. The cited contexts embrace the period between the 34^th^ and the 29^th^ century BC. Similarities are found both with long endscrapers, as shown by the examples from Zug-Vorstadt [139], Arbon Bleiche 3 (Thurgau) [92], Steinhausen-Sennweid (Zug) [113–114] and Twann-Bahnhof (Bern) [114] (Fig 28, 2,4 and 6–7), and with instruments defined as “blades with modified extremities” or “knives” exemplified by the tools (Fig 28, 5,8 and 11) from Feldmeilen-Vorderfeld (Zürich) [140], Ermatingen-Westerfeld (Thurgau) [141] and Pfäffikon Burg (Zürich) [142]. It cannot be excluded that some had been hafted as the typical “Horgen knives”, which are fist-knives with lateral convex wood or bark handles. In general, the resharpening of the lateral edges was a common practice of blade tools used as knives [142]. The fact that knives could, after their use on siliceous plants, be equipped with an endscraper front as is the case with the Iceman’s tool, is attested at Zürich Parkhaus Opéra [143] and at Arbon Bleiche 3 [92] (Fig 28, 9–10).

Ötzi’s endscraper-knife shows fitting comparisons to the Northern Alpine Horgen tradition, where multifunctional blade tools, used among others for cutting siliceous plants, were part of the personal equipment for everyday use.

## 6. Discussion and conclusions

### 6.1 From the chert outcrops to the final user

The interdisciplinary research on the Iceman chert tools and retoucher corrects and integrates previous results allowing new insights into Ötzi‘s individual history and his last days, as well as to the way of life of alpine Copper Age communities during the latter part of the 4^th^ millennium BC.

The cherts of the small lithic assemblage - six artefacts possessed by the Iceman at the moment of his violent death - originate from at least three different areas of provenance of the Southern Alpine domain (Fig 22). The Scaglia Variegata Alpina of arrowhead 12 was collected in the Non Valley outcrops in Trentino, an area yielding abundant and good quality cherts exploited at least since the Mesolithic [43, 46]. Lying at about 40 km in a direct line from the Vinschgau, the valley where Ötzi‘s community is thought to have lived, the Non Valley outcrops represent indeed the nearest exploitable cherts. High altitude routes leading from here northwards, reaching the Lower Vinschgau and the area of Meran, have been reconstructed for the Mesolithic [144] and could have still been in use during Ötzi‘s time. A possible cultural convergence between the Non Valley and the Vinschgau is suggested by the *statuae stele* of both territories which display a similar striped pattern on the back [33].

The Maiolica of the Iceman dagger was probably collected in the south-western Trentino, roughly 60–75 km from the Vinschgau, but even more south-western provenances cannot be completely excluded (eastern Lombardy Prealpine foothills between lakes Garda and Iseo). The prehistoric chert exploitation of this area, palaeogeographically lying on the margin of the Lombard Basin, is currently under study [145]. The resources of the south-western Trentino could have been accessed by the Copper Age communities peopling the area of the lower Sarca valley in the East and also to the Valcamonica communities settled in the West. Finally, the provenance of the Maiolica from arrowhead 14, endscraper, borer and small flake cannot be established in detail. Comparable varieties spread over several chert-bearing areas of the Trento Plateau, in the actual Trentino and Veneto, including the Baldo-Bondone area, lying at least 70 km from the Vinschgau, the plateaus of Folgaria and Asiago, the Monti Lessini and the Grappa Mountains.

The variety of cherts represented even by the scarce lithic artefacts found by the Iceman, suggests an extensive provisioning and circulation network on the Southern slope of the Alps which is not at all limited to the often cited Monti Lessini. Alpine communities without locally available raw material were supplied directly, or by itinerant knappers, by high-quality cherts arriving from different extraction and production centers.

Regarding the modalities the raw material arrived, we have to consider that at the time of the Iceman, lithic production of certain objects was presumably a specialized activity to a certain degree, especially for blade blanks, arrowheads and daggers. Considerable time is needed for developing the necessary skill to become a specialized flint knapper, and, once having gained a high degree of workmanship, a single knapper could easily supply one or even several villages. For this reason we can suppose that Ötzi was not himself the maker of the blades and the blanks his tools are made from. In addition, his equipment did not comprise the necessary working tools - neither a punch to produce blades nor an organic hammer to produce flake blanks and preforms–instruments that a specialized flintknapper would carry. It is more likely that people like Ötzi had occasionally or periodically the opportunity to obtain blade blanks or at least blade tools with functional potential. The fact that Ötzi‘s blade tools - endscraper and borer - are made from two different Maiolica varieties, might indicate that he obtained them from intermediary persons and possibly one by one. Regarding Ötzi‘s supply of flake blanks or preforms, an interesting hint is given by the small flake. Being a flaking waste from the production of foliates (arrowheads or daggers), and therefore not an object which circulates in a trade network, Ötzi could have collected it in the workshop or waste of a specialized knapper with the intention to use it.

In its small way the Iceman’s toolkit reflects the usual composition of the eneolithic industries characterized by few functional types, namely bifacial tools, projectile points, multipurpose blade tools and expedient tools on by-products [138]. The typological character of the assemblage displays influences of both Southern and Northern tradition. The first aspect is best evidenced by the numerous arrowheads comparable to the Iceman tanged projectile points in use among the different cultural groups of Northern Italy. In contrast, the Iceman’s endscraper, formerly a knife, is reminiscent of the characteristic blade tools of the Eastern Swiss Horgen culture. Considering that the Vinschgau is equidistant from the Eastern Swiss lakes and from the Po River, such analogies will not be a surprise in the toolset of an Alpine man. In fact, convergences in the material culture between the settlement Isera la Torretta (Trento) and sites of the Swiss Canton Grisons led to the definition of the transalpine cultural group “Tamins-Carasso-Isera 5” at about 3500-3000/2900 cal BC, contemporary with the Swiss Horgen and the Southern German Cham culture [131, 146]. The permeability of the Alps is best exemplified by the above cited exportation of bifacial lithic daggers which shows the intensive transalpine contacts. The wide circulation of such tools could be the reason why the Iceman’s notched dagger finds more similarities with distant daggers than with more local ones.

### 6.2 What tools tell us about the owner

Even if Ötzi was not the producer of the blanks for his tools, he was used to doing flintworking. He possessed a fully functional antler retoucher which shows evident wear of use. The pressure retouch displayed on five lithic tools, in some cases freshly made, is compatible with such an antler point retoucher. It seems therefore very realistic to say that Ötzi retouched and resharpened his own tools, as most probably most adults did. The common level of pressure skill on his tools appears as quite homogeneous and can be evaluated as medium to good (level of 6/7 on a scale of 1 to 10), which signifies a level of skill that is just effective enough to achieve correct results in retouching and resharpening.

Ötzi‘s tools display wear traces from use. They were mainly used for plant processing: cutting reeds or soft wood with the small flake, cutting siliceous plants with both endscraper edges in a prolonged action and occasionally with one edge of arrowhead 14. Sickle gloss is not an uncommon feature on lateral edges of long endscrapers. At tomb 27 of Le Mose (Piacenza) [147], dated to 4386±50 BP (3323–2898 cal BC) a long endscraper used as a “plant knife” and deposited with a male adult, suggests that such cutting tools were part of the personal equipment. On the other hand, it cannot have been usual at Ötzi‘s time to cut siliceous plants with an arrowhead. The arrow point with gloss found at Arbon Bleiche 3 at Lake Constance [92] is interpreted as the recycling of a former sickle. Points with gloss from Pre-Pottery and Pottery Neolithic sites in Northern Syria and Southeast Turkey are interpreted as the expedient use or as re-utilization of tools [148]. Considering the importance for an archer of having a functioning arrow, and the risk of damage by its improper use, an expedient utilization is most probable also for arrow 14. It is remarkable that Ötzi did not use his dagger for cutting plants, although in terms of tool efficiency flint daggers are very functional for such use. Cutting and scraping vegetal material is attested on two daggers from Ala in Trentino [149], whereas cutting siliceous plants was one of several actions carried out with the flint dagger from Allensbach at Lake Constance [150]. Also the Final Neolithic Grand Pressigny daggers from France are mostly used for cutting Gramineae [151]. To think of it, the dagger was not essential in the Iceman’s toolkit. All actions–cutting, scraping, boring and even striking a light if necessary–could be done with the smaller and lighter tools contained in the belt pocket. Was the role of the dagger restricted to specific tasks and mainly symbolic? Based on the deposition in the graves and their representation on the anthropomorphic *stelae*, daggers appear in fact as status symbols of adult males of the Copper Age [89, 152]. Hence, without wanting to deny a functional role of the Iceman’s dagger, the item could have represented a distinctive sign for the social identity of its owner. Keeping it in a dedicated scabbard equipped with a leather eyelet, Ötzi could indeed have carried it in view, fixed on an outer belt.

While it is possible that Ötzi carried his dagger in view, matters are different for the retoucher. In order to assure a good functioning it has to be kept in a dry place, because humidity decreases the hardness of the antler point. A good way to preserve the tool would have been to carry it in the belt pouch, as Ötzi did with the fungus. At the moment of discovery, though, the retoucher was found near or under the mummy. If the Iceman was not retouching when he died (in fact all the lithic implements were put away in their bags except for the dagger which we don’t know), we could hypothesize that the pressure flaker was put away in some pocket of his coat, or that it was fixed on a belt worn under the coat.

### 6.3 The question of the foreign arrow

Based on the different manufacture of the two finished arrowheads carried in the quiver, Paulsen [153] and Egg and Spindler (see p. 203 in [9]) have proposed that “most probably they had been produced by two different persons”, suggesting that “one if not both arrowheads might not have been made by Ötzi but recovered by him, maybe after having been shot off by his pursuers” (p. 113). Examining the technical features of both arrows in detail, the hypothesis that they were not produced by the same person is consistent also with the new results (Table 2).

**Table 2:** Comparison of the two finished arrowheads.

	Arrow 12	Arrow 14
PRODUCTION		
Shaft wood	*Viburnum lantana* and *Cornus* sp.	*Viburnum lantana*
Shaft technology	shaft with inserted foreshaft	shaft with distal thickening
Shaft length, total	87–88 cm	about 81 cm
Shaft surface	dark marks	not attested
Feather fletching	winding to the right	winding to the left
Chert raw material	Scaglia Variegata Alpina	Maiolica
Provenance area	Non Valley	Trentino or Veneto (Trento plateau)
Retouch	made by pressure	made by pressure
Shape	tang & continuous profile	tang & with distinct shoulders
Typology	not common	very common
Birch-bark-tar	small dots on surface	large spots on surfaces, “messy”
USE & REPAIR		
Resharpenings	Resharpened, at least once	Resharpened, at least twice
Pressure retouch	Careful shaping, but not systematic	Careful shaping, but not systematic
Edges	sharp	sharp
Tip	Broken and not repaired	Freshly resharpened
Use wear	Only contact (?)	Cutting siliceous plants, “improper”
LAST STATE		
Tar cover on shaft	largely eliminated	intact
Arrowhead breakage	broken at the center	tang proximally broken
Tang fragment	found still hafted	not found
Potential repair of arrowhead	possible	no need, still usable
Shaft preservation	proximal fracture, pieces nearly *in situ*	mesial fracture, pieces nearly *in situ*
Feather fletching	pieces missing	fewer pieces missing

Data are based on the present research and integrated with results from former studies [9, 16, 64]. Differently from what is reported in the literature, the neat breaks of shafts 12 and 14, which lay nearly in primary position inside the quiver, are here interpreted as post-depositional.

All aspects related to the production phase are in fact significantly different. Regarding the authorship, the Iceman was not the maker of arrow 14. Ötzi was a right-hander, which is proved by the incisions on his retoucher tip and the sickle gloss on the edges of his endscraper-knife, whereas the feather fletching of arrow 14 shows a winding to the left. On the other hand, there is no argument against the hypothesis that Ötzi himself was the producer of arrow 12 with the fletching that winds to the right. This hypothesis would be in line with the more local chert from the near Non Valley, combined with a more local, alpine arrowhead type. Furthermore, the shaft length of arrow 12 is identical to 9 out of the 11 unfinished but complete shafts (see Fig 54 in [9]). Although he was not the maker, Ötzi could have already possessed arrow 14 for some time: the two arrowheads show a similar style in pressure retouching, which suggests that the resharpenings were made by the same person.

### 6.4 Ötzi’s last days

During his last itinerary Ötzi carried along a limited kit of lithic tools, light and just sufficient for the essential tasks. The different raw materials and production modes indicate that they came into his possession at different moments. All of them display traces of a rather long tool-life being in a very advanced techno-economic state:

- a resharpened small dagger with a broken tip;
- an almost worn out blade tool, intensely used as a knife and freshly resharpened at its distal end;
- a freshly retouched borer, from a previous retouched blade;
- no dedicated strike-a-light, but a possible expedient use of the endscraper and point for such purpose;
- only one small flake, though very sharp and easy to carry, instead of having a handful of them;
- only two arrowheads (already resharpened one or more times) both with broken tang but no flake blanks for making new ones, despite possessing a functioning retoucher.

**Fig 29:**
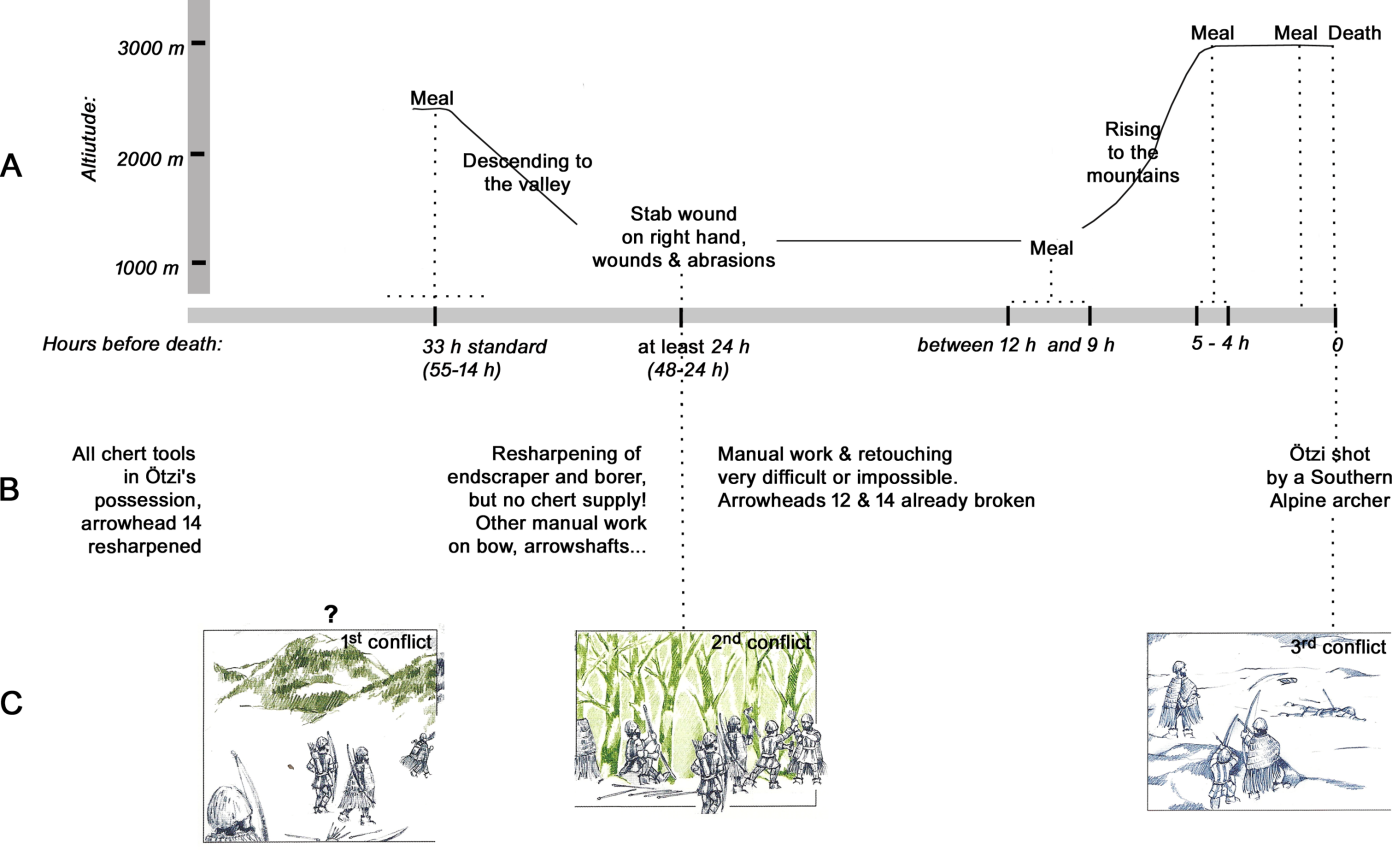
Results of the present study integrated to past reconstructions of Ötzi‘s last days, based on the Iceman last itinerary and meals [154, 158–160], the state of his wounds [155–156], the causes of his death [157] and the damaged and insufficient equipment [9].

This means that Ötzi had not had access to chert of any kind for quite some time, neither from outcrops nor from a specialized flint-knapper workshop. How does this information match the reconstruction of Ötzi’s last days? Analyses of the background pollen contained in the food residuals of the mummy’s digestive tract have allowed to trace the Iceman’s itinerary during his last two days by deducing the environments In which the meals were eaten [154]. Roughly 33 hours before his death (possible variations are between 14–55 hours) he stayed in the alpine/subalpine range at about 2,500 m a.s.l., north or south of the main Alpine watershed (Fig 29A). From here he descended to 1,200 m a.s.l. on the southern slope of the Alpine ridge where he spent some time and from where he departed again to reach the mountains above 3,000 m a.s.l. 4–5 hours before his death. During the time lapse of 48–24 hours before his death the Iceman suffered a stab wound on his hand [155–156]. Its position, between thumb and index finger, and its depth are typical for self-defense. These data, together with the damaged and insufficient equipment and clothing—in particular the unfinished bow and arrows—as well as the Iceman’s violent death far from his village [157] led Egg and Spindler to speculate about Ötzi‘s involvement in a series of conflicts [9] (Fig 29C). The first conflict was hypothesized in order to explain the absence of an operative bow and arrows from his equipment with their loss or damage (see Fig 120 in [9]).

Trying to integrate the data of the present research to the time-based sequence and reconstructed scenario put forward by the cited authors, the following observations can be made (Fig 29B): It is very likely that the entire toolkit under study here, including arrow 14, already resharpened in the same style of arrowhead 12, was in Ötzi‘s possession before the hectic itinerary of his two last days. The time of the breakage of the two lithic arrowheads cannot be stated, but could have happened during these hours. One can reasonably assume that the last resharpenings of the chert tools, the fresh retouch of endscraper and borer planned for future manual work, were carried out by Ötzi before he suffered the deep wound on his right hand, which must have been disabling for a right-hander. The injury could also be considered as the *terminus ante quem* for any other manual work he had in progress: the finishing of the bow blank made from a yew trunk, the finishing of the arrowshafts from *Viburnum lantana* shoots as well as other repair. But, as it seems, this was not the only impediment he had to face: although having an urgent need, he did not obtain any new lithic arrowheads or chert blanks to make these. Evidently though having descended to lower altitudes, he did not have access to a village or to persons to acquire the necessary items. Maybe for this reason Ötzi might have kept the broken arrowheads, and also took some antler points with him, an alternative raw material for arrowhead manufacture.

The Iceman was therefore in a critical situation when he returned to the mountains hours later and reached the Tisenjoch. The lethal arrow which hit him from behind, shot by a Southern Alpine archer, was therefore only the last in a series of difficulties he had to face, and which also left traces in his lithic toolkit.
